# Finetuning Hole-Extracting Monolayers for Efficient Organic Solar Cells

**DOI:** 10.1021/acsami.2c01900

**Published:** 2022-03-30

**Authors:** Haijun Bin, Kunal Datta, Junke Wang, Tom P. A. van der Pol, Junyu Li, Martijn M. Wienk, René A. J. Janssen

**Affiliations:** † Molecular Materials and Nanosystems & Institute for Complex Molecular Systems, Eindhoven University of Technology, Eindhoven 5600 MB, The Netherlands; ‡ Dutch Institute for Fundamental Energy Research, Eindhoven 5612 AJ, The Netherlands

**Keywords:** organic solar cells, hole-transport layer, monolayer, 3PACz, nonfullerene acceptor

## Abstract

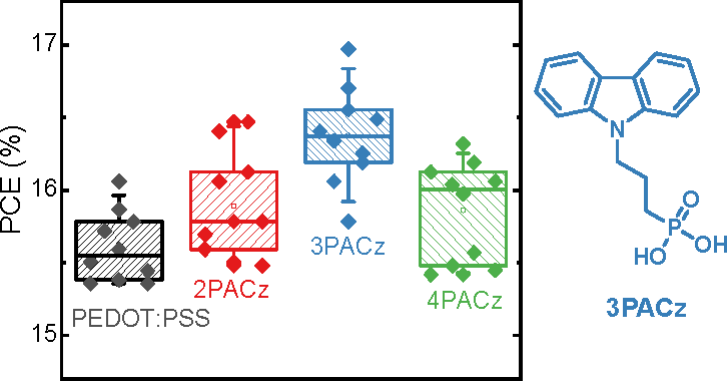

Interface layers used for electron transport (ETL) and hole transport (HTL) often significantly enhance the performance of organic solar cells (OSCs). Surprisingly, interface engineering for hole extraction has received little attention thus far. By finetuning the chemical structure of carbazole-based self-assembled monolayers with phosphonic acid anchoring groups, varying the length of the alkane linker (2PACz, 3PACz, and 4PACz), these HTLs were found to perform favorably in OSCs. Compared to archetypal PEDOT:PSS, the PACz monolayers exhibit higher optical transmittance and lower resistance and deliver a higher short-circuit current density and fill factor. Power conversion efficiencies of 17.4% have been obtained with PM6:BTP-eC9 as the active layer, which was distinctively higher than the 16.2% obtained with PEDOT:PSS. Of the three PACz derivatives, the new 3PACz consistently outperforms the other two monolayer HTLs in OSCs with different state-of-the-art nonfullerene acceptors. Considering its facile synthesis, convenient processing, and improved performance, we consider that 3PACz is a promising interface layer for widespread use in OSCs.

## Introduction

Organic solar cells (OSCs) attract the interest of the photovoltaic community because of their rapidly improving power conversion efficiency (PCE) combined with characteristic advantages, including potential low cost, ink-based large-area production, light weight, flexibility, and semitransparency.^1−5^ In most OSCs, the photovoltaic active layer is sandwiched between a transparent conductive oxide front electrode and a metal back electrode. Interface layers between the active layer and the electrodes are generally used to improve the selective extraction of photogenerated electrons and holes.^6−10^ Recent efforts have focused mainly on developing novel polymer donors and nonfullerene acceptors and have resulted in astoundingly high PCEs of 17–19%,^11−22^ which are almost on par with commercial photovoltaic technologies. Next to the photoactive layer, interface layers, such as the electron-transport layer (ETL) and the hole-transport layer (HTL), can contribute significantly to enhancing device performance.^19,23^ However, interface engineering, especially the development of new HTLs, has received comparatively little attention.^18,24,25^

Interface layers are carrier-selective charge-transport layers that help generate a built-in electric field and a barrier-free contact between the electrode and photoactive layer, allowing charge carriers to drift and be collected.^26,27^ Most efforts have so far focused on studying ETLs; many alcohol-soluble ETLs, including PFNBr, PNDIT-F3N, PDINO, PDINN (for systematic names, see the Supporting Information), and others, have been developed and are widely used in high-efficiency OSCs.^6,7,26−28^ Interestingly, some small-molecule-based solar cells do not require an ETL to reach high efficiency.^28,29^ In contrast, HTL material development has lagged behind. Despite reports of impressive inorganic composites and nanosheets as HTL to create high-performance OSCs, their complex preparation methods seem to hinder widespread use and commercialization.^23,24,30^ Poly(3,4-ethylene-dioxythiophene):poly(styrenesulfonate) (PEDOT:PSS) is by far the most ubiquitous HTL used in OSCs and has become the standard. However, the modest conductivity, corrosive and hygroscopic properties, and absorption in the near-infrared region of PEDOT:PSS may restrict performance improvements and compromise the long-term stability.^24,30^ As a result, there is a need for novel solution-processable HTLs with optimal characteristics and potential for commercial application.

Hole-extracting monolayers have recently emerged as a valuable strategy to improve the performance of metal-halide perovskite solar cells. Albrecht et al. developed and advanced various hole-selective self-assembled monolayer forming agents such as 2PACz, MeO-2PACz, and Me-4PACz for high-performance perovskite solar cells.^31^ These materials form a monolayer on indium tin oxide (ITO) electrodes via the anchoring phosphonic acid moiety.^32^ Perovskite solar cells that use these undoped monolayers as HTL provide PCEs over 20% and are potentially cost-effective due to extremely low material consumption and scalable processing. These materials are also suitable for more complex device configurations such as tandem solar cells because they provide a conformal coverage on rough metal oxide textures.^33^ Monolithic metal-halide perovskite-silicon tandem solar cells using Me-4PACz as a hole contact material have achieved a PCE of 29.15%,^31^ and copper indium gallium selenide (CIGSe)-perovskite tandem solar cells using MeO-2PACz afford a PCE of 23.26% at a cell area of 1.03 cm^2^.^34^ Encouraged by these excellent results, researchers also incorporated hole-extracting monolayers in OSCs and obtained PCEs over 18%.^18,19,21,35^ Despite these promising examples, hole-extracting monolayers have attracted limited attention in OSCs. The examples above also demonstrate that the chemical structure can have a significant influence on the performance, implying that finetuning of the molecular design is critical in obtaining novel monolayer molecules for efficient OSCs.

**Figure 1:**
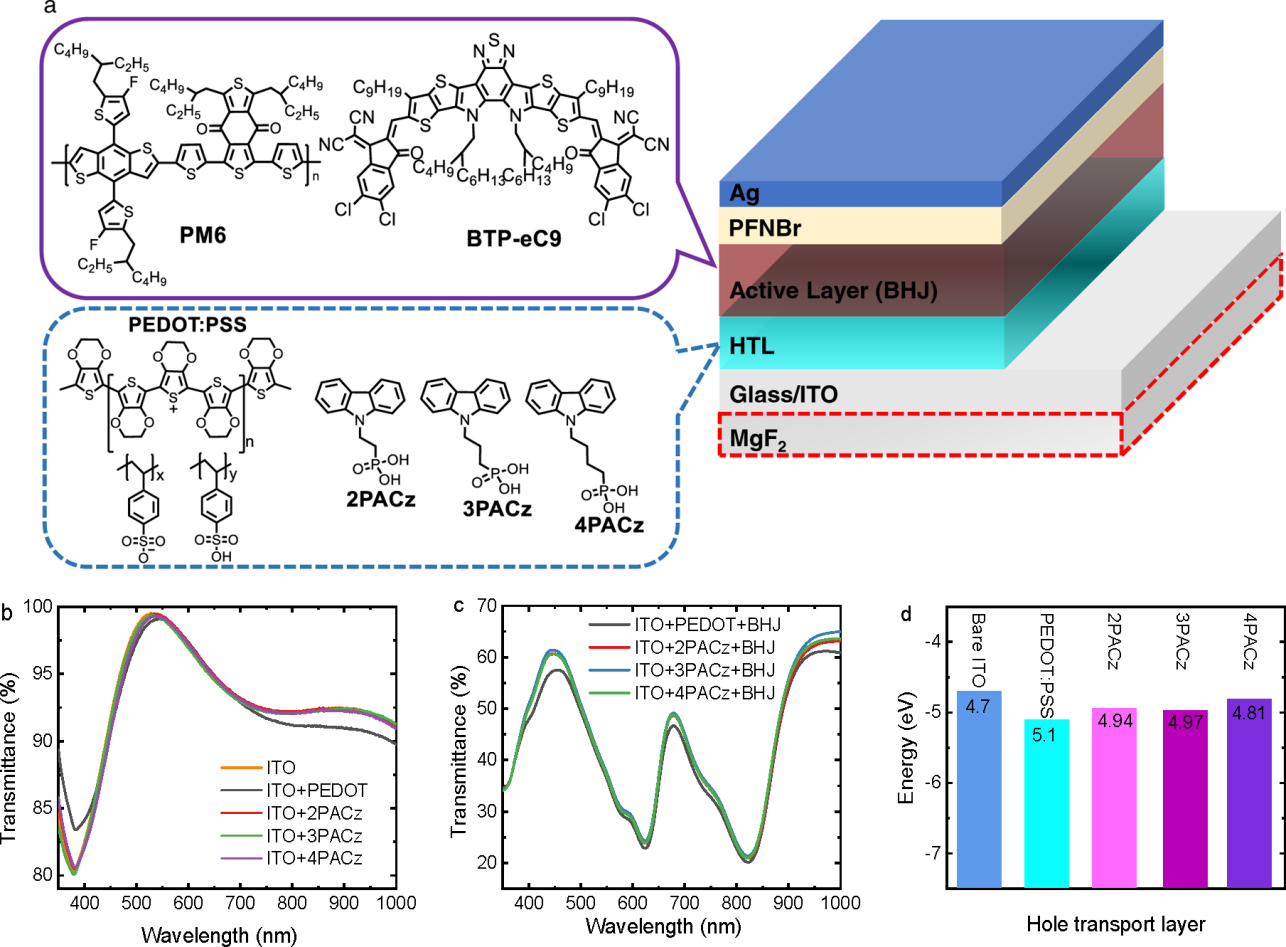
(a) Device structure of the OSCs and the chemical structures of active layer materials and HTLs. (b) Transmittance spectrum of bare ITO and HTL-covered ITO electrodes. (c) Transmittance spectrum of the BHJ layers on glass/ITO covered with HTLs. (d) Schematic energy-level diagram showing the work functions of bare ITO and ITO covered with PEDOT:PSS, 2PACz, 3PACz, and 4PACz.

Herein, we designed 3PACz (Figure 1), a carbazole-based molecule with phosphonic acid as the anchoring group. Its chemical structure is similar to 2PACz,^18^ but it has one more methylene group in the side chain. Two closely related molecules, 2PACz and 4PACz, and diluted PEDOT:PSS as a reference, were used as the HTL to examine the impact molecules with very similar structures but subtle differences in alkyl chains on device performance. All three molecules can be easily processed into self-assembled monolayers without any post-treatment. Importantly, the PACz monolayers exhibit higher optical transmittance and reduce series resistance compared to PEDOT:PSS layers, leading to improved device performance. Among the monolayer-based OSCs, 3PACz-based devices show the best performance, with fill factors approaching 0.80, and provide PCEs of 17.4% with excellent reproducibility for different nonfullerene acceptors. The results demonstrate that 3PACz is a promising alternative to PEDOT:PSS for high-performance and cost-effective OSC devices.

## Results and Discussion

Figure 1a shows the device structure, the structures of PM6 and BTP-eC9^20^ used in the bulk-heterojunction (BHJ) photoactive layer, and the chemical structures of the HTLs. 2PACz is commercially available. The synthesis of 3PACz and 4PACz is depicted in Scheme S1 and the molecules were obtained in high yield via three simple reactions. Inexpensive raw materials and short and high-yield preparations make 3PACz cost-effective. The details on the synthesis and molecular characterization are described in the Supporting Information. 2PACz and 3PACz have good solubility in methanol and ethanol. The solubility of 4PACz is slightly lower, and solutions of 4PACz are slightly turbid at the same concentrations. All three molecules are insoluble in water and in weakly polar organic solvents such as chloroform and chlorobenzene. This enables layer-by-layer solution processing of conventional devices.

PACz molecules were dissolved in ethanol at a concentration of 1.0 mmol mL^–1^ and then spin coated directly onto ultraviolet-ozone treated ITO electrodes on glass at 4000 rpm. The phosphonic acid group forms strong and stable bonds to ITO allowing for assembly of robust monolayers.^19,27,34,36^ As a reference, a PEDOT:PSS layer with a thickness of approximately 20 nm was spin coated onto the ITO electrodes using the commercial PEDOT:PSS dispersion diluted with deionized water (v/v, 1:1).^37^ Transmittance spectra (Figure 1b) indicate that the thin PEDOT:PSS film reduces the transmittance of light in the 420–550 nm and 700–1000 nm wavelength ranges compared to ITO, whereas the PACz monolayers cause minimal changes in transmittance. A similar trend is found after casting the PM6:BTP-eC9 photoactive layer on these HTLs (Figure 1c). Ultraviolet photoelectron spectroscopy (UPS) was performed to determine the work function of the monolayer-functionalized ITO-electrodes (Figure S1). The work functions are 4.94, 4.97, and 4.81 eV for 2PACz, 3PACz, and 4PACz, respectively, and are slightly lower than the 5.1 eV work function of PEDOT:PSS on ITO. The reduced work functions did not affect the open-circuit voltage (*V*_oc_) of the OSCs (vide infra).

**Figure 2:**
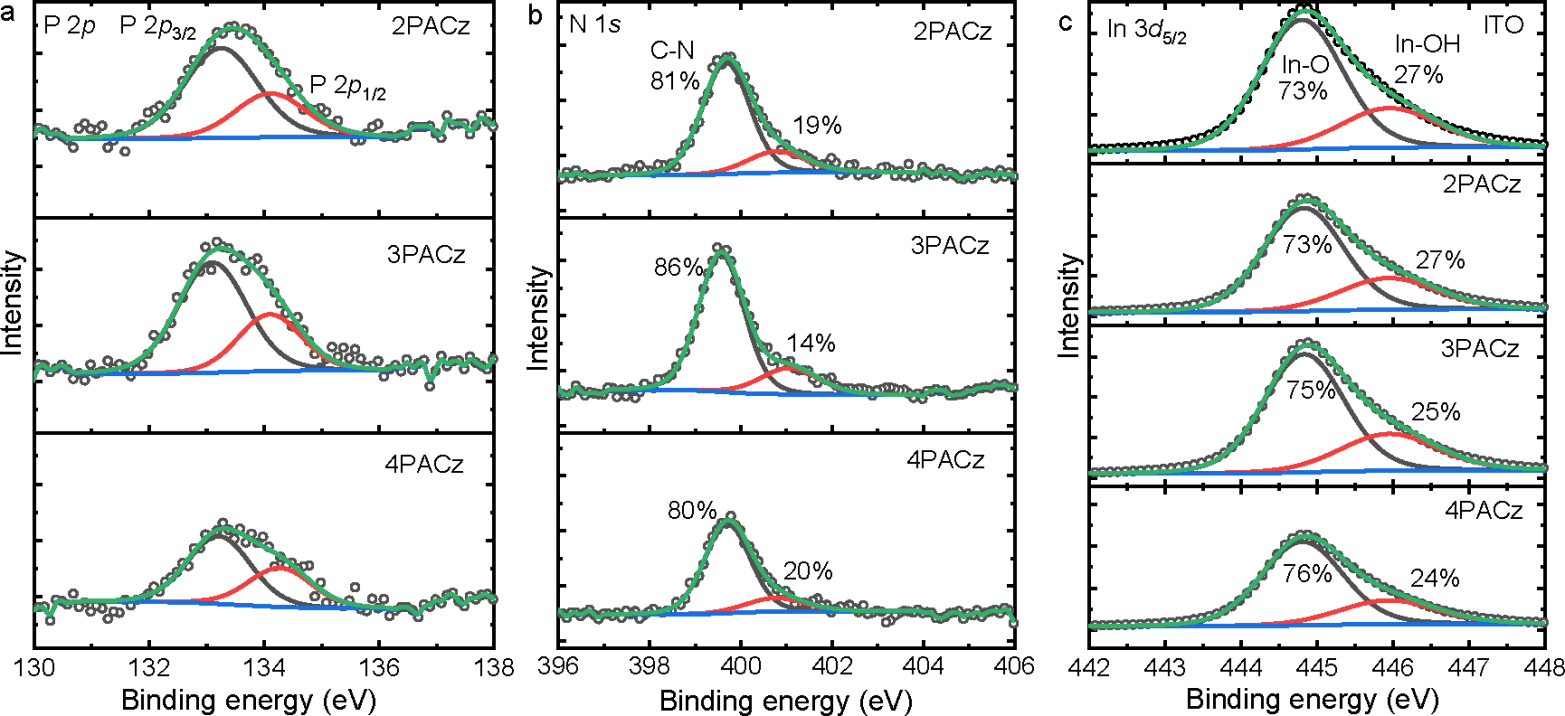
High-resolution XPS scans of the atomic core levels for ITO surface without and with monolayers on top. (a) P 2p, (b) N 1s, and (c) In 3d.

To confirm the presence of the monolayers on the ITO surface, we performed X-ray photoelectron spectroscopy (XPS). Figure 2 shows high-resolution XPS scans of ITO without and with the monolayers. For the monolayers, signals characteristic of N 1s and P 2p electrons can directly be attributed to the presence of the PACz molecules. Because XPS is surface sensitive, probing up to an average depth of approximately 5 nm,^25,38^ In 3d and Sn 3d signals originating from the ITO electrode can also be observed in the XPS spectra of the monolayers on ITO. These results confirm the ultrathin nature of the monolayer HTLs. The atomic concentrations of characteristic atoms (N, P for monolayers and In, Sn for ITO) are summarized in Table S1. The surface morphologies of bare ITO and the HTL films were investigated by tapping-mode atomic force microscopy (AFM). Figure S2 shows the height and phase images of the ITO surface without and with HTLs. The three PACz monolayers give rise to root-mean-squared surface roughness (*R*_q_) close to 1.0 nm, and similar to that of bare ITO (*R*_q_ = 0.8 nm). Furthermore, the phase images reveal that ITO and monolayers display similar surface textures. In contrast, the 20 nm PEDOT:PSS layer presents a smaller *R*_q_ of 0.55 nm and larger surface domains than the other four surfaces. These results indicate that monolayers do not change the surface features of ITO due to the ultrathin nature, unlike PEDOT:PSS that generates a new smooth surface. The surface height histograms extracted from the AFM images (Figure S3) show a smoother PEDOT:PSS surface due to the narrower height distribution, while the PACz monolayers have height distributions comparable to bare ITO.^18^ Hence, both XPS and AFM indicate that the PACz molecules form monolayers on ITO.

The performance of the hole-transporting monolayers was explored by fabricating conventional-configuration glass/ITO/HTL/PM6:BTP-eC9/PFNBr/Ag OSCs (Figure 1a). PACz molecules were dissolved in ethanol at 1.0 mmol mL^–1^, and then spin coated onto the ITO electrodes at 4000 rpm for 60 s, without any post-treatment to form monolayers, optimization of specific processing conditions are summarized in Table S2 and Figure S4. Further details on the device fabrication are provided in the Supporting Information. OSCs with hole-transporting monolayers provide PCEs close to 16%, i.e., higher than for the PEDOT:PSS-based device with a PCE of 15.5% (Figure S5 and Table S3). The enhanced PCEs result from increased *J*_sc_ and FF. The 3PACz-based OSC has the highest performance. Although the work functions of the monolayers on ITO are lower than those of PEDOT:PSS on ITO, the open-circuit voltage (*V*_oc_ = 0.86 V) is the same in each case, implying that the hole-transporting monolayers form an ohmic contact with the active layer without causing additional *V*_oc_ losses.

**Figure 3:**
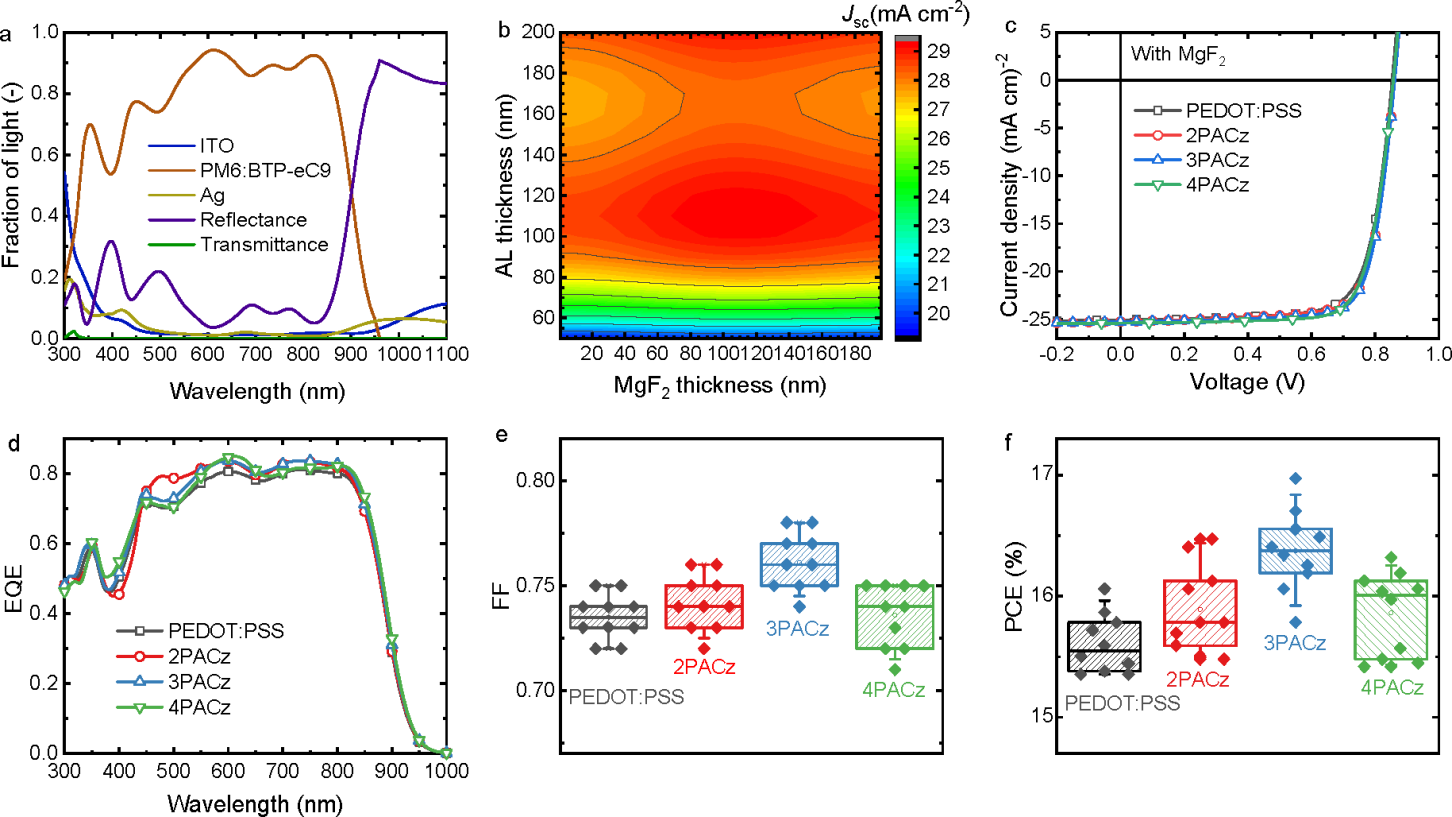
(a) Optically simulated reflectance and absorptance spectra of the layers in an OSC with a PACz HTL. (b) Optical simulations of *J*_sc_ as a function of the thicknesses of the active and MgF_2_ layers for OSC with a PACz HTL assuming IQE = 100%. (c) *J*–*V* characteristics of PM6:BTP-eC9 OSCs (with MgF_2_) for different HTLs). (d) Corresponding EQE spectra. (e) Statistical distribution of FF. (f) Statistical distribution of PCE.

One reason for the moderate performance of the control device on ITO/PEDOT:PSS is its relatively low *J*_sc_.^19,20,39^ Optical simulations using the transfer matrix method on the entire device stack (Figure 3a) reveal that considerable photon loss is caused by reflection of light from the front glass surface. Introducing a MgF_2_ (*n* ≈ 1.4) antireflection coating is effective in enhancing transmission of light though the glass (*n* > 1.5).^40^ Optical simulations (Figure 3b) reveal that a 105 nm MgF_2_ antireflection layer combined with a 110 nm active layer result in an optimum *J*_sc_ over 29.0 mA cm^–2^, when assuming 100% internal quantum efficiency (IQE). Accordingly, OSCs with a MgF_2_ layer provide an enhanced *J*_sc_ (Figure 3c, d and Table 1) compared to the same devices without MgF_2_ (Figure S5 and Table S3). As a result, the PCE of the control device with PEDOT:PSS increases to 16.1%. Similarly, the PCEs of devices with the monolayers increased to 16.5% (2PACz), 17.0% (3PACz), and 16.3% (4PACz).

**Table 1:** Photovoltaic Parameters of PM6:BTP-eC9 OSCs with Different HTLs

HTL	*J*_sc_^a^ (mA cm^–2^)	*V*_oc_ (V)	FF^a^	PCE^a^ (%)	*J*_sc_^b^ (mA cm^–2^)	PCE^b^ (%)
PEDOT:PSS	24.9 (24.69 ± 0.137)	0.86	0.75 (0.735 ± 0.011)	16.1 (15.61 ± 0.243)	25.04	16.2
2PACz	25.2 (24.90 ± 0.205)	0.86	0.76 (0.742 ± 0.013)	16.5 (15.89 ± 0.358)	25.84	16.9
3PACz	25.3 (25.02 ± 0.155)	0.86	0.78 (0.761 ± 0.014)	17.0 (16.38 ± 0.335)	25.91	17.4
4PACz	25.3 (25.06 ± 0.171)	0.86	0.75 (0.736 ± 0.015)	16.3 (15.86 ± 0.334)	25.80	16.6

a Values from *J–V* measurement.

b Values from EQE measurement. The data outside the brackets are the highest value, and the data inside the brackets are the average value calculated from 10 individual solar cells.

When comparing PEDOT:PSS and PACz devices, 2PACz and 3PACz monolayers provide a higher FF. This is reflected by the shunt (*R*_sh_) and series (*R*_s_) resistances.^41,42^ The *R*_sh_ is 4.5, 5.0, 5.5, and 4.8 kΩ cm^2^ for PEDOT:PSS, 2PACz, 3PACz, and 4PACz, respectively, whereas *R*_s_ is 18.6, 17.7, 18.0, and 17.8 Ω cm^2^. The monolayers thus result in higher *R*_sh_ and lower *R*_s_ than PEDOT:PSS, consistent with the improved FF.^43^*R*_s_ is not only determined by charge transport in the photoactive layer but also by the contribution of the HTL. The latter can be estimated from the current–voltage (*I–V*) curves of ITO/HTL/Ag devices (Figure S6)^30^ and compared to that of ITO/Ag as a reference. The resistance was 1.17 Ω cm^2^ for bare ITO, and 1.38, 1.19, 1.17, and 1.23 Ω cm^2^ for PEDOT:PSS, 2PACz, 3PACz, and 4PACz HTLs. Clearly, the monolayer-based devices have almost unchanged resistances compared to that of bare ITO, whereas even a thin PEDOT layer results in a small increase in *R*_s_.

External quantum efficiency (EQE) spectra (Figure 3d and Figure S4b) reveal a higher response in the 300–920 nm wavelength range when using MgF_2_. The *J*_sc_ of OSCs without/with MgF_2_ integrated from the EQE spectra are 24.0/25.0, 24.4/25.8, 24.6/25.9, and 24.4/25.8 mA cm^–2^ for the PEDOT:PSS, 2PACz, 3PACz, and 4PACz devices, respectively, and agree well with the values obtained from the solar simulator. Optical simulations (Figure S7) confirm the increase in *J*_sc_ when going from PEDOT:PSS to a PACz monolayer as HTL. The monolayers improve *J*_sc_ compared to PEDOT:PSS because parasitic absorption is reduced, but experimental differences in *J*_sc_ among the three PACz molecules are small. The MgF_2_ antireflection layer increases *J*_sc_ of the PACz monolayer devices by ∼1.4 mA cm^–2^ compared to 1.0 mA cm^–2^ for the PEODT:PSS device. The statistical distribution of the FF and PCE reveals that monolayers result is slightly wider spread than PEDOT:PSS but still demonstrate good reproducibility (Figure 3e, f, Table 1). Overall, the trend in PCE resembles the difference in FF and all the monolayer-based solar cells surpass the efficiency of PEDOT:PSS cells, with 3PACz yielding the highest efficiency of 17.0% in the *J–V* scan and 17.4% after the EQE correction.

To test the wider applicability of hole-transporting monolayers, we have tested OSCs based on two more blends, PM6:Y6-BO-4Cl^20^ and PM6:Y6-BO-4F,^44^ with PEDOT:PSS and PACz monolayers (Figures S8 and S9 and Tables S4 and S5). Both blends exhibit the same trend; with PEDOT:PSS, the PCEs are lower than 16%, whereas the monolayer-based devices have PCEs >16% because of the higher *J*_sc_ and FF. Among the monolayers, 3PACz gave again the highest FFs, approaching 0.80, implying that 3PACz performs very well with state-of-the-art OSCs.

**Figure 4:**
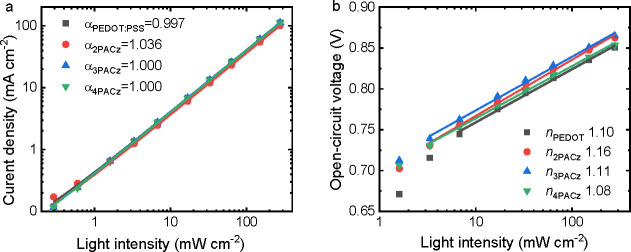
(a) Light intensity dependence of *J*_sc_. (b) Light intensity dependence of *V*_oc_. In fitting the *V*_oc_, we ignored the measurements at the lowest light intensity because the *V*_oc_ is most affected by residual shunts.

Charge recombination was studied by measuring the light intensity (*P*_light_) dependence of *J*_sc_ and *V*_oc_. The slopes of log *J*_sc_ vs log *P*_light_ are very close to or equal to 1 (Figure 4a) and imply negligible bimolecular recombination at short-circuit. The slope of a plot of *V*_oc_ vs ln *P*_light_ is close to the thermal energy (*kT*/*q*, where *k* is the Boltzmann constant, *T* is the absolute temperature, and *q* is the elementary charge) for all cells (Figure 4b), suggesting trap-assisted recombination is suppressed. To gain more insight into the effect of the monolayer HTL on the photovoltaic performance, the charge collection efficiency of the four OSCs was estimated using the photocurrent density (*J*_ph_ = *J*_light_ – *J*_dark_) as determined from the *J–V* characteristics in the dark and under illumination. An indication of the efficiency of charge collection can obtained by dividing *J*_ph_ at short circuit (0 V) by the saturated photocurrent density (*J*_sat_) at −2 V. This resulted in 95.8%, 98.2%, 98.5%, and 98.8% for PEDOT:PSS, 2PACz, 3PACz, and 4PACz based devices respectively, indicating that the monolayers improve collection efficiency, contributing to the high FF.

**Figure 5:**
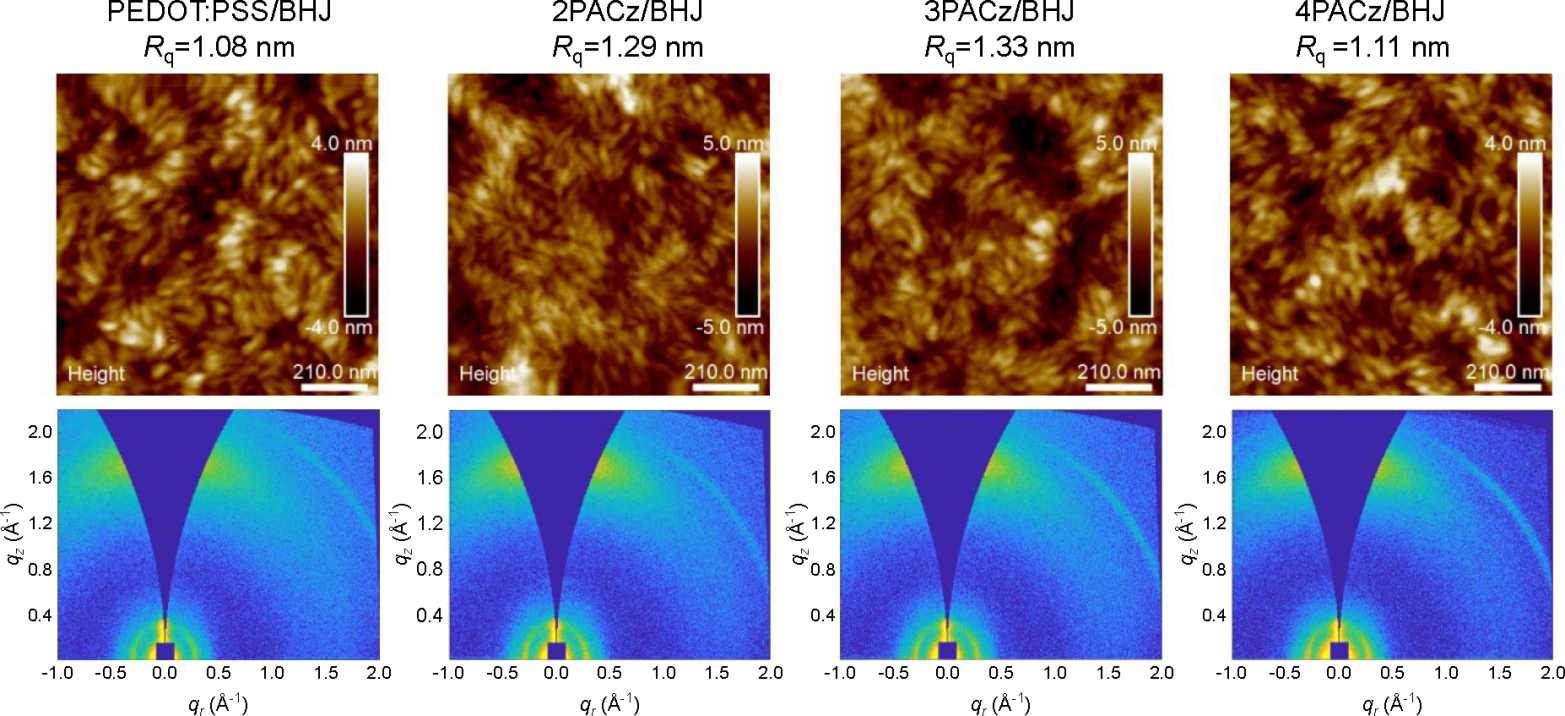
AFM height images of the active layer cast on the top of four HTLs (top) and the corresponding 2D GIWAXS patterns (bottom).

Considering that charge transport is also affected by the nanoscale phase separation and molecular packing of the donor and acceptor, the morphology of BHJ films cast on top of four HTLs was studied in more detail. First, the surface morphologies of the four BHJ films were investigated by AFM in tapping-mode (Figure 5). The AFM height images reveal that the four films have a similar *R*_q_ (root-mean-squared surface roughness) of approximately 1.1 nm and exhibit well-distributed fibrous structures, indicating a smooth and ordered surface structure for all films. Two-dimensional grazing-incidence wide-angle X-ray scattering (2D GIWAXS) measurements were conducted to investigate the molecular packing of the films (Figure 5). The corresponding in-plane and out-of-plane line-cut profiles are shown in Figure S10. Except for the subtle differences in the intensity of diffraction peaks that may be caused by the difference in film thickness, all four films show nearly identical 2D GIWAXS patterns with a preferred face-on orientation. The peaks in the out of plane and in plane directions are at ∼1.74 and ∼0.29 Å^–1^, respectively, consistent with those reported in the literature.^20^ Accordingly, we infer that there is no difference in the morphology of the active layer prepared on these four different HTLs. This implies that the improvement of the performance is not related to a change in morphology.

High-resolution XPS measurements of the Cl 2p and F 1s binding energy regions for the four blends are shown in Figure S11 (Supporting Information) and the atomic concentrations are summarized in Table S6 (Supporting Information). Cl and F are the tracer atoms for BTP-eC9 and PM6. The very similar intensity of Cl 2p and F 1s signals and atomic concentrations confirms that the four active layers have the same distribution of donor and acceptor and thus the same morphology.

In principle, the surface free energy of the hole-transport layer can affect the morphology of the active layer.^45^ To investigate surface energies, the water contact angles for the three PACz layers were measured (Figure S12). The water contact angle is marginally lower for 3PACz (71.8°) than for 2PACz (73.6°) and somewhat higher for 4PACz (83.9°). For PEDOT:PSS the water contact angle is a function of time: it starts above 70° but decreases with time.^46^ Hence, water contact angles and thus surface energies do not strongly differ for the hole-transport layers, consistent with the similar blend morphologies found by AFM and GIWAXS (Figure 5).

The combined results show the PACz layers and especially 3PACz are interesting alternatives to PEDOT:PSS for use in OSCs. Next to performance, stability is an important issue for OSCs. So far, two studies comparing the OSC stability using 2PACz monolayers give rather different results, and suggest that more extensive studies are needed to resolve the differences.^18,21^

## Conclusions

Through finetuning the chemical structure of carbazole-based self-assembled monolayers, by varying the length of the alkyl linker between the carbazole unit and the surface-anchoring phosphonic acid group, we found that 3PACz shows superior performance when used as a monolayer HTL in OSCs, compared to 2PACz, 4PACz, and PEDOT:PSS. XPS and AFM measurements demonstrated that monolayers were successfully integrated onto the top of the ITO electrode. Compared to PEDOT:PSS, all PACz monolayer HTLs exhibit higher optical transmittance and lower electrical resistance, which is beneficial for the *J*_sc_ and FF improves. Among the three monolayer-based devices, the 3PACz-based device consistently provides the best performance using PM6 and three different nonfullerene acceptors (BTP-eC9, Y6-BO-4Cl, and Y6-BO-4F) giving the highest FF approaching 0.80. Using an MgF_2_ antireflection layer, 17.4% PCE has been realized in PM6:BTP-eC9 OSCs with 3PACz as monolayer HTL. This is distinctively higher for a reference device (16.2%) with PEDOT:PSS as HTL. AFM, 2D-GIWAXS, and XPS revealed that the PACz monolayer HTLs do not affect the morphologies of the BHJ active layer. Considering its facile synthesis and convenient processing, we think that 3PACz can also be applied successfully in semitransparent OSCs and has promising potential for commercialization.

## Experimental Section

Chemical reagents and catalysts were purchased from Sigma-Aldrich and used as received, unless otherwise specified. PM6, BTP-eC9, Y6-BO-4Cl, and Y6-BO-4F were purchased from Solarmer Materials Inc. (Beijing, China). 2PACz was purchased from TCI. The detailed experimental and fabricated conditions are shown in the Supporting Information.

## Supporting Information Available

Experimental details of the synthesis of 3PACz and 4PACz, device fabrication details, characterizations of the OCSs (i.e., measurements and instruments used), UPS, XPS, AFM, GIWAXS, and ^1^H NMR and mass spectra (PDF)
