# Supplementary material for: Finetuning Hole-Extracting Monolayers for Efficient Organic Solar Cells
Source: ACS Appl Mater Interfaces. 2022 Mar 30;14(14):16497–504. doi: 10.1021/acsami.2c01900 (PMC9011343; doi:10.1021/acsami.2c01900)
Supplement: Supplementary file 1 — am2c01900_si_001.pdf [file am2c01900_si_001.pdf]

Supporting Information

Finetuning Hole-Extracting Monolayers for Efficient Organic Solar Cells

Haijun Bin,[1] Kunal Datta,[1] Junke Wang,[1] Tom P. A. van der Pol,[1] Junyu Li,[1] Martijn M. Wienk,[1] and René A. J. Janssen*[1,2]

[1] *Molecular Materials and Nanosystems & Institute for Complex Molecular Systems, Eindhoven University of Technology, Eindhoven, 5600 MB, The Netherlands.*

[2] *Dutch Institute for Fundamental Energy Research, Eindhoven, 5612 AJ, The Netherlands*

* corresponding author, E-mail: r.a.j.janssen@tue.nl

Full names of compounds used and mentioned in the main text

2PACz	(2-(9*H*-carbazol-9-yl)ethyl)phosphonic acid
3PACz	(3-(9*H*-carbazol-9-yl)propyl)phosphonic acid
4PACz	(4-(9*H*-carbazol-9-yl)butyl)phosphonic acid
BTP-eC9	2,2'-[[12,13-bis(2-butyloctyl)-12,13-dihydro-3,9-dinonylbisthieno[2",3":4',5']thieno[2',3':4,5]pyrrolo[3,2-*e*:2',3'-*g*][2,1,3]benzothiadiazole-2,10-diyl]bis[methylidyne(5,6-chloro-3-oxo-1*H*-indene-2,1(3*H*)-diylidene)]]bis[propanedinitrile]
Me-4PACz	[4-(3,6-dimethyl-9*H*-carbazol-9-yl)butyl]phosphonic acid
MeO-2PACz	(2-(3,6-dimethoxy-9*H*-carbazol-9-yl)ethyl)phosphonic acid
PM6	poly[[4,8-bis[5-(2-ethylhexyl)-4-fluoro-2-thienyl]benzo[1,2-*b*:4,5-*b*']dithiophene-2,6-diyl]-2,5-thiophenediyl[5,7-bis(2-ethylhexyl)-4,8-dioxo-4*H*,8*H*-benzo[1,2-*c*:4,5-*c*']dithiophene-1,3-diyl]-2,5-thiophenediyl])
PDINN	*N*,*N*'-bis{3-[3-(dimethylamino)propylamino]propyl}perylene-3,4,9,10-tetracarboxylic diimide
PDINO	*N*,*N*'-bis(*N*,*N*-dimethylpropan-1-amine oxide)perylene-3,4,9,10-tetracarboxylic diimide
PFNBr	poly(9,9-bis(3'-(*N*,*N*-dimethyl)-*N*-ethylammoinium-propyl-2,7-fluorene)-alt-2,7-(9,9-dioctylfluorene))dibromide
PNDIT-F3N	poly[[2,7-bis(2-ethylhexyl)-1,2,3,6,7,8-hexahydro-1,3,6,8-tetraoxobenzo[*lmn*][3,8]phenanthroline-4,9-diyl]-2,5-thiophenediyl[9,9-bis[3-(dimethylamino)propyl]-9*H*-fluorene-2,7-diyl]-2,5-thiophenediyl]
Y6-BO-4Cl	(2,2'-((2*Z*,2'*Z*)-((12,13-bis(2-butyloctyl)-3,9-diundecyl-12,13-dihydro-[1,2,5]thiadiazolo[3,4-*e*]thieno[2",3":4',5']thieno[2',3':4,5]pyrrolo[3,2-*g*]thieno [2',3':4,5]thieno[3,2-*b*]indole-2,10-diyl)bis(methanylylidene))-bis(5,6-dichloro-3-oxo-2,3-dihydro-1*H*-indene-2,1-diylidene))dimalononitrile))
Y6-BO-4F	(2,2'-((2*Z*,2'*Z*)-((12,13-bis(2-butyloctyl)-3,9-diundecyl-12,13-dihydro-[1,2,5]thiadiazolo[3,4-*e*]thieno[2",3":4',5']thieno[2',3':4,5]pyrrolo[3,2-*g*]thieno [2',3':4,5]thieno[3,2-*b*]indole-2,10-diyl)bis(methanylylidene))-bis(5,6-difluoro-3-oxo-2,3-dihydro-1*H*-indene-2,1-diylidene))dimalononitrile))

Experimental Section

Materials. Chemical reagents and catalysts were purchased from Sigma Aldrich. PM6, BTP-eC9, Y6-BO-4Cl, and Y6-BO-4F were purchased from Solarmer Materials Inc. (Beijing, China). 2PACz was purchased from TCI. 3PACz, 4PACz were synthesized according to the procedure reported in the literature.[1]

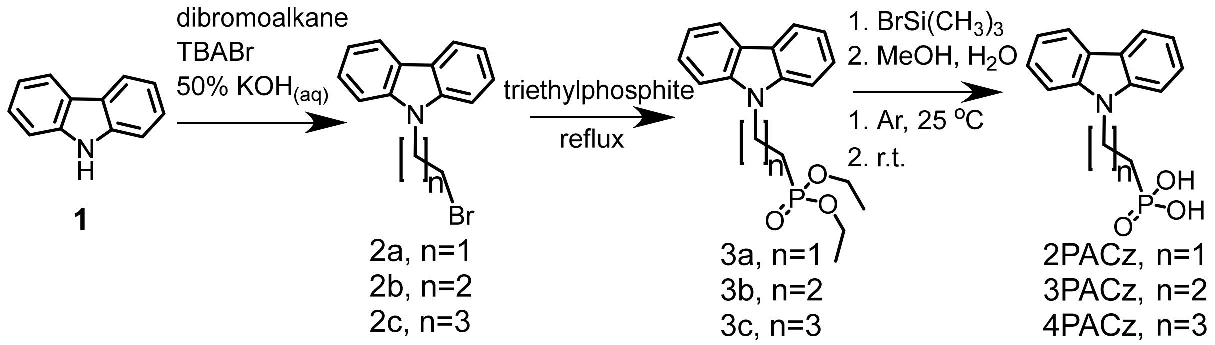

Scheme S1. The synthetic routes for 3PACz and 4PACz.

9-(3-Bromopropyl)-9*H*-carbazole (2b). 9H-carbazole (**1**) (1.67 g, 10.0 mmol) was dispersed in toluene (20 mL). Afterwards, catalytic amount of tetrabutylammonium bromide, 50% KOH aqueous solution (26 mL), and 1,3-dibromopropane (2.04 mL, 20.0 mmol) were added. After 24 h stirring at room temperature, an additional amount of 1,3-dibromopropane (2.04 mL, 20.0 mmol) was added, and the mixture was stirred at room temperature for additional 24 h. Then the mixture extracted with ethyl acetate, the organic layer was dried over anhydrous $MgSO_4$ and the solvent was distilled off under reduced pressure. The product was purified by column chromatography (*n*-hexane), to give 2.02 g (70 %) of white solid. ^1H NMR (400 MHz, $CDCl_3$): δ 8.11-8.09 (d, 2H), 7.48-7.47 (m, 4H), 7.26-7.25 (m, 2H), 4.52-4.49 (t, 2H), 3.40-3.37 (t, 2H), 2.46-2.42 (m, 2H). GC-MS calculated for $C_{15}H_{14}BrN$ *m/z* = 287.03, 289.03. Found *m/z* = 287, 289.

Diethyl (3-(9*H*-carbazol-9-yl)propyl)phosphonate (3b). Compound **2b** (1.44 g, 5.0 mmol) was dissolved in triethyl phosphite (15 mL, 87.7 mmol) and the reaction mixture was heated at reflux for 24 h. After reaction completion the solvent was distilled off under reduced pressure. The crude product was purified by column chromatography (*n*-hexane; acetone:*n*-hexane, 1:4, v:v) to give 1.61 g (93%) of colorless viscous liquid. ^1H NMR (400 MHz, CD_3OD): δ 8.15-8.13 (d, 2H), 7.66-7.64 (d, 2H), 7.48-7.44 (t, 2H), 7.23-7.19 (t, 2H), 4.58-4.55 (t, 2H), 4.07-3.98 (m, 4H), 2.18-2.10 (m, 2H), 1.84-1.76 (m, 2H), 1.30-1.21 (m, 6H). GC-MS calculated for $C_{19}H_{24}NO_3P$ *m/z* = 345.15. Found *m/z* = 345.

(3-(9*H*-Carbazol-9-yl)propyl)phosphonic acid (3PACz). Compound **3b** (1.34 g, 3.88 mmol) was dissolved in anhydrous 1,4-dioxane (30 mL) under argon atmosphere and bromotrimethylsilane (4.5 mL, 34.1 mmol) was added dropwise. Reaction was stirred for 24 h at 25 °C under argon atmosphere. Afterwards solvent was partially distilled off under reduced pressure, and the liquid residue was dissolved in methanol (30 mL). Next, distilled water was added dropwise (50 mL), until solution became opaque. Product was filtered off and washed with water to give 715.2 mg (64 %) of white solid. ^{1}H NMR (400 MHz, $(CD_3)_2SO$): δ 8.16-8.14 (d, 2H), 7.66-7.64 (d, 2H), 7.47-7.43 (m, 2H), 7.22-7.18 (t, 2H), 4.50-4.46 (t, 2H), 2.01-1.94 (m, 2H), 1.58-1.49 (m, 2H). MALDI-TOF MS calculated for $C_{19}H_{24}NO_3P$ *m/z* = 289.09. Found *m/z* = 288.08 (M−H).

9-(4-Bromobutyl)-9*H*-carbazole (2c). The procedure is identical to that of **2b** but using 1,4-dibromobutane instead of 1,3-dibromopropane**.** ^{1}H NMR (400 MHz, $CDCl_3$): δ 8.15-8.13 (d, 2H), 7.61-7.59 (d, 2H), 7.48-7.44 (d, 2H), 7.23-7.19 (d, 2H), 4.52-4.49 (t, 2H), 3.55-3.52 (t, 2H), 2.82-2.78 (m, 2H), 2.04-1.95 (m, 2H). GC-MS calculated for $C_{16}H_{16}BrN$ *m/z* = 301.05, 303.05. Found *m/z* = 301, 303.

Diethyl (4-(9*H*-carbazol-9-yl)butyl)phosphonate (3c). The procedure is identical to that of **3b** but using **2c** instead of **2b.** ^{1}H NMR (400 MHz, CD_3OD): δ 8.14-8.13 (d, 2H), 7.61-7.59 (d, 2H), 7.47-7.43 (t, 2H), 7.22-7.18 (t, 2H), 4.47-4.44 (t, 2H), 3.98-3.94 (m, 4H), 2.01-1.97 (m, 2H), 1.77-1.65 (m, 4H), 1.28-1.17 (m, 6H). GC-MS calculated for $C_{20}H_{26}NO_3P$ *m/z* = 359.17. Found *m/z* = 359.

(4-(9*H*-Carbazol-9-yl)butyl)phosphonic acid (4PACz). The procedure is identical to that of **3PACz** but using **3c** instead of **3b.** ^{1}H NMR (400 MHz, $(CD_3)_2SO$): δ 8.09-8.08 (d, 2H), 7.56-7.54 (d, 2H), 7.40-7.37 (m, 2H), 7.15-7.11 (m, 2H), 4.35-4.32 (t, 2H), 1.81-1.77 (m, 2H), 1.51-1.46 (m, 4H). MALDI-TOF MS calculated for $C_{16}H_{18}NO_3P$ *m/z* = 303.10. Found *m/z* = 302.10 (M−H).

Material characterization. Transmittance was recorded on a PerkinElmer Lambda 1050 UV-vis-near IR spectrophotometer. HTL films were prepared by spin coating solutions on indium tin oxide (ITO) covered glass substrates. Ultraviolet photoelectron spectroscopy (UPS) was carried out in a multi-chamber ESCALAB II system using He-I radiation (E_{He-I}=21.22 eV) and a –6 V bias. The samples were deposited on glass substrates fully covered by ITO and transferred into the vacuum chamber directly from N_2 atmosphere. X-ray photoelectron spectroscopy (XPS) was performed using a Thermo Scientific K-Alpha with a 180° double focusing hemispherical analyzer and a 128-channel detector. Monochromatic Al Kα (1486.6 eV) radiation was used, and the X-ray spot size was 400 μm. For the surface analysis, a survey spectrum was first measured for 15 scans with a pass energy of 200 eV. High-resolution scan (30 times) of each element was conducted with a pass energy of 50 eV. The surface morphologies of films were characterized by a Dimension 3100 atomic force microscope in tapping mode.

Device fabrication. The OSCs were fabricated and characterized in a N_2-filled glovebox. Pre-structured ITO-coated glass substrates were cleaned in acetone and isopropyl alcohol for 10 min each. After drying, (for the MgF_2 based device, we deposited the MgF_2 in vacuum first) the substrates were UV-ozone treated for 30 min. Then coated with poly(3,4-ethylenedioxythiophene) polystyrene sulfonate (PEDOT:PSS) (Heraeus, Clevios P VP Al 4083, diluted with deionized water v/v = 1:1, 20 nm) or the monolayers. The active layer was spin coated in a N_2-filled glovebox from a solution of PM6:BTP-eC9 with total concentration of 16 mg mL^{-1} in CB. After spin-coating, the active layer was SVA for 1 min by CS_2. Then a solution of PFNBr in methanol at a concentration of 0.5 mg mL^{-1} was deposited on the active layer. Finally, a top Ag electrode was deposited in vacuum at a pressure of about 5.0×10^{-7} Pa. The cells with an area of 0.09 cm^2 were masked with a size of 0.0676 cm^2.

GIWAXS. Grazing incidence wide angle X-ray scattering (GIWAXS) experiments were

carried out on a GANESHA 300 XL+ system from JJ X-ray equipped with a Pilatus 300K detector (pixel size 172 µm × 172 µm). The X-ray source is a Genix 3D Microfocus Sealed Tube X-Ray Cu-source with integrated Monochromator (multilayer optic "3D version" optimized for SAXS) (30 W) and the wavelength used was λ = 1.5408 Å. The sample was placed vertically on the goniometer and tilted to a glancing angle of 0.2° with respect to the incoming beam. A small beam was used to get a better resolution. The primary slits have a size of 0.3 × 0.5 mm^2, and the guard slits have a size of 0.1 × 0.9 mm^2. The accumulation time was 2 h for each measurement.

Optical-Electrical Modeling. Optical simulations based on the transfer matrix method were performed using Setfos 5.0 (FLUXiM). The wavelength dependent refractive index – extinction coefficient (n−k) spectra of the different layers used in the simulations were determined from the reflectance corrected transmission (PM6:BTP-eC9),[2] variable angle spectroscopic ellipsometry (VASE) using a WVASE31 ellipsometer (J.A. Woollam Co.) (MgF_2), taken from the Setfos database (PEDOT:PSS AL4083), or from the literature (ITO[3], glass[4], and silver[5]). The monolayers and PFNBr layer are very thin (few nanometers) and were omitted from the simulations. The n−k spectra used are shown in the graph below (Figure S0).

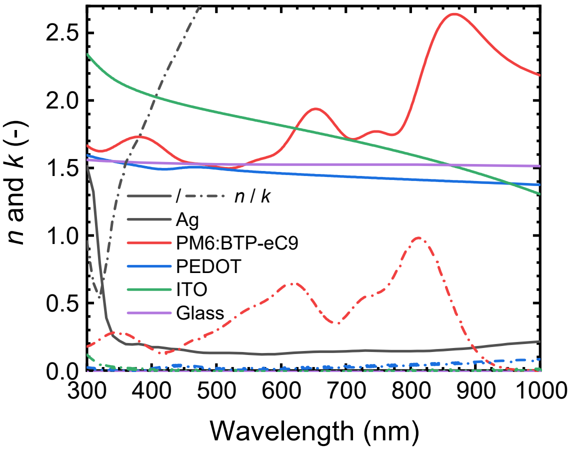

Figure S0. n−k spectra of all materials in the stack used in the optical simulations

Additional Figures and Tables

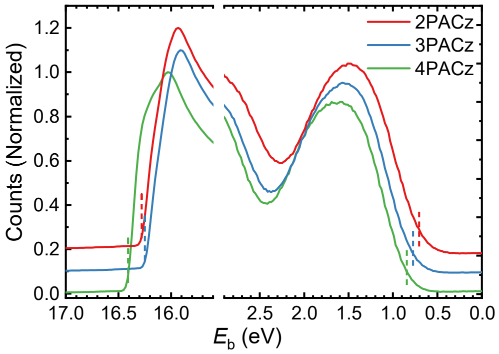

Figure S1. UPS spectra of 2PACz, 3PACz, and 4PACz-covered ITO electrodes. Spectra are offset vertically for clarity.

Table S1. Atomic concentration determined by XPS for in different layers.

Sample	C 1*s* (%At conc.)	N 1*s* (%At Conc.)	O 1*s* (%At Conc.)	P 2*p*1 (%At Conc.)	In 3*d*5 (%At Conc.)	Sn 3*d*5 (%At Conc.)
ITO	25.45	-	48.36	-	23.52	2.67
ITO+2PACz	33.74	2.44	38.95	6.02	17.02	1.82
ITO+3PACz	38.21	2.95	35.47	7.09	14.75	1.54
ITO+4PACz	25.30	1.70	40.1	2.3	28.8	1.70

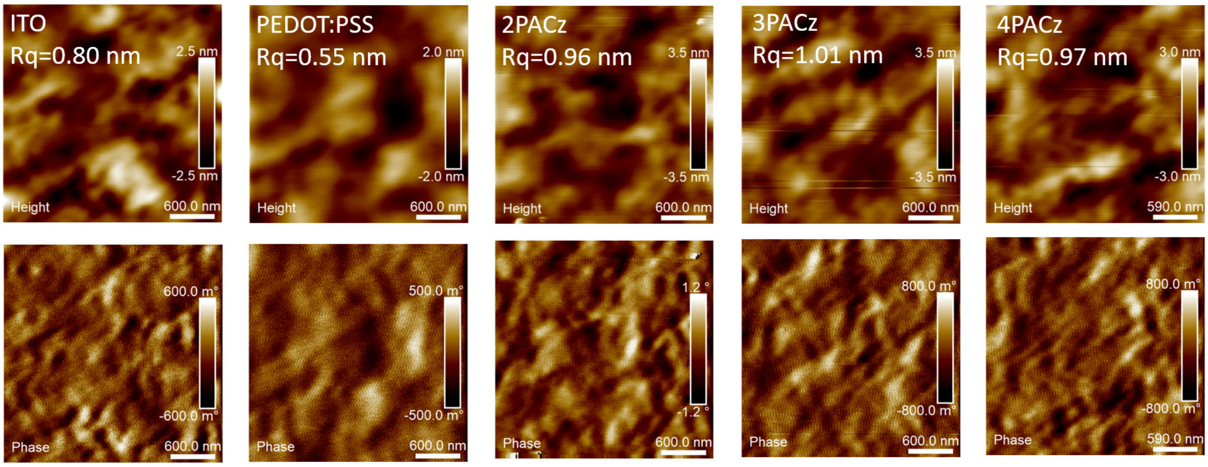

Figure S2. AFM Height (top) and phase images (bottom) of ITO, ITO/PEDOT:PSS, and ITO-2/3/4PACz films with scan areas of 3 μm × 3 μm.

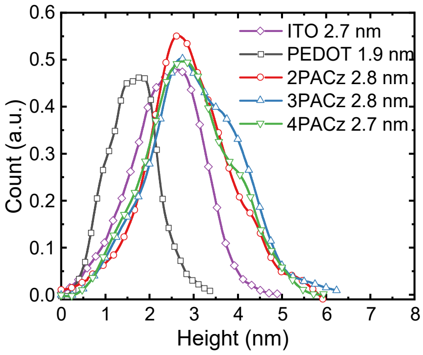

Figure S3. Surface height histograms of ITO, ITO/PEDOT:PSS, and ITO-2/3/4PACz films obtained by AFM.

Table S2. Photovoltaic parameters of the OSCs based on PM6:BTP-eC9 with 3PACz as HTL under various condition.

Concentration (mmol/mL)	Solvent	Speed (rpm)	Annealed	J_{sc} (mA cm^{-2})	V_{oc} (V)	FF	PCE (%)
1.5	EtOH	4000	no	24.1	0.83	0.65	13.1
1.0	EtOH	4000	no	25.0	0.85	0.72	15.2
0.5	EtOH	4000	no	25.4	0.80	0.70	14.6
1.0	EtOH	4000	no	24.3	0.86	0.75	15.7
1.0	MeOH	4000	no	24.3	0.86	0.73	15.2
1.0	IPA	4000	no	23.4	0.85	0.74	14.7
1.0	EtOH	3000	no	24.6	0.86	0.75	15.8
1.0	EtOH	4000	no	24.6	0.86	0.76	16.0
1.0	EtOH	5000	no	24.5	0.86	0.76	16.0
1.0	EtOH	4000	100 °C 10 min	24.8	0.86	0.75	16.0

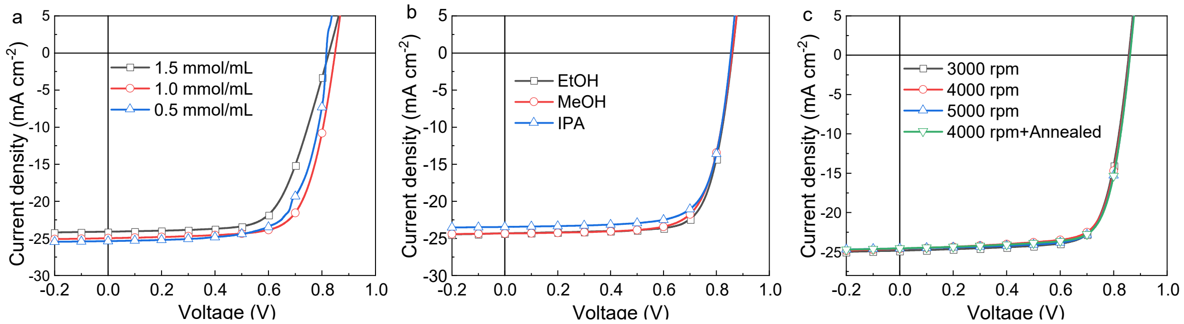

Figure S4. *J-V* curves of PM6:BTP-eC9-based OSCs with 3PACz as HTL deposited under various conditions. Standard conditions are a concentration of 1.0 mmol/mL in EtOH and 400 rpm. (a) Variation in concentration. (b) Variation in solvent. (c) Variation in spin speed and effect of thermal annealing.

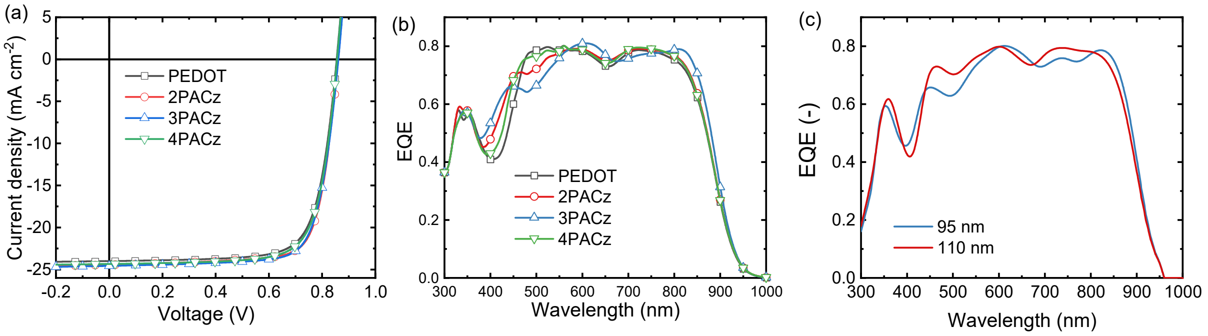

Figure S5. (a) *J-V* curves of PM6:BTP-eC9-based OSCs with various HTLs without MgF_2. (b) corresponding EQE spectra. (c) Optical simulations showing that the small differences in EQE between 2PACz and 4PACz versus 3PACz are explained by small differences (110 vs. 95 nm) in the thickness of the PM6:BTP-eC9 layer.

Table S3. Photovoltaic parameters of the OSCs based on PM6:BTP-eC9 without MgF_2.

Blend	J_{sc} (mA cm^{-2})	V_{oc} (V)	FF	PCE (%)
PEDOT:PSS	24.0 (24.0)	0.86	0.75	15.5 (15.5)
2PACz	24.4 (24.4)	0.86	0.76	16.0 (16.0)
3PACz	24.6 (24.6)	0.86	0.77	16.1 (16.1)
4PACz	24.3 (24.4)	0.86	0.75	15.7 (15.7)

The data in brackets are obtained from EQE measurement.

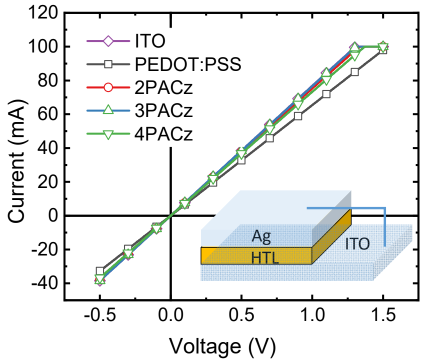

Figure S6. *I-V* characteristics of conductivity measurements for bare ITO, PEDOT:PSS and the monolayers.

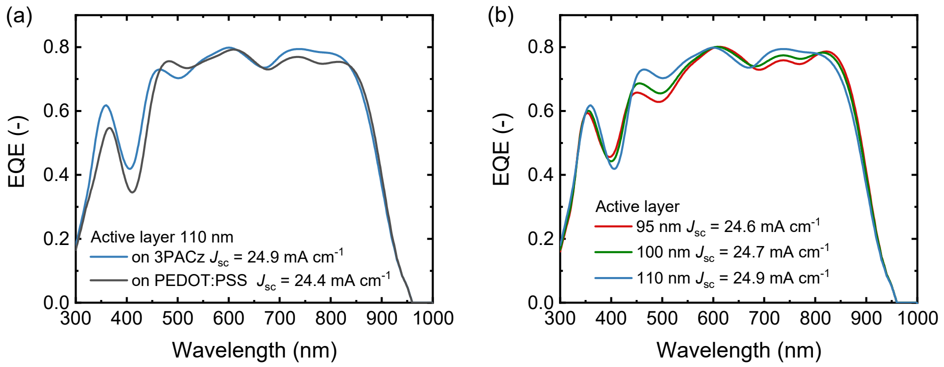

Figure S7. (a) Optical simulated EQEs for solar cells with PEDOT:PSS (30 nm) and 3PACz HTLs for a 110 nm active layer, assuming IQE = 85%. (b) Same for 3PACz and three different active layer thicknesses. Integrated short-circuit current densities are given in the insets.

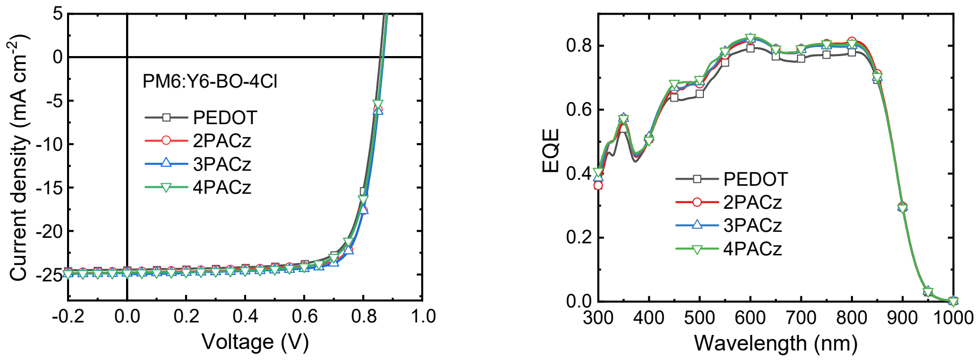

Figure S8. *J-V* curves of PM6:Y6-BO-4Cl-based OSCs with various HTLs and the corresponding EQE spectra.

Table S4. Photovoltaic parameters of OSCs based on PM6:Y6-BO-4Cl.

Blends	J_{sc} (mA cm^{-2})	V_{oc} (V)	FF	PCE (%)
PEDOT:PSS	24.4 (24.1)	0.86	0.75	15.7 (15.5)
2PACz	24.7 (25.0)	0.87	0.77	16.6 (16.7)
3PACz	24.9 (24.9)	0.87	0.78	16.9 (16.9)
4PACz	24.8 (25.1)	0.87	0.76	16.4 (16.6)

The data in brackets are obtained from EQE measurement.

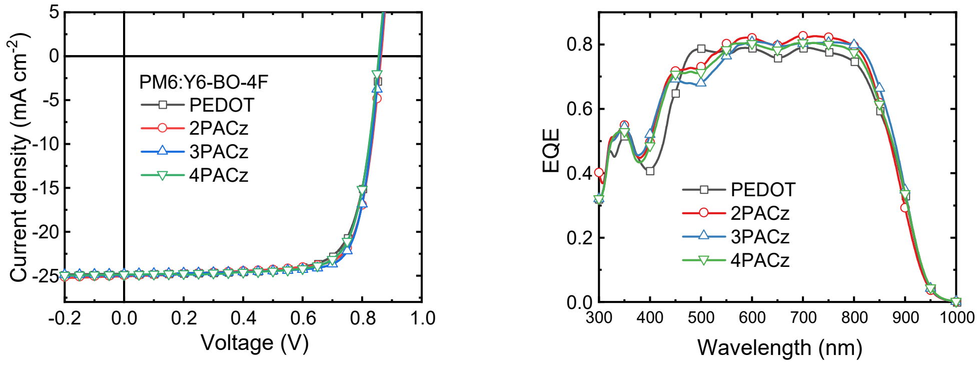

Figure S9. *J-V* curves of PM6:Y6-BO-4F-based OSCs with various HTLs and the corresponding EQE spectra.

Table S5. Photovoltaic parameters of OSCs based on PM6:Y6-BO-4F.

Blends	J_{sc} (mA cm^{-2})	V_{oc} (V)	FF	PCE (%)
PEDOT:PSS	24.8 (24.3)	0.85	0.75	16.0 (15.5)
2PACz	24.9 (25.2)	0.86	0.78	16.6 (16.9)
3PACz	24.8 (25.0)	0.86	0.79	16.8 (17.0)
4PACz	24.9 (24.8)	0.86	0.76	16.3 (16.2)

The data in brackets are obtained from EQE measurement.

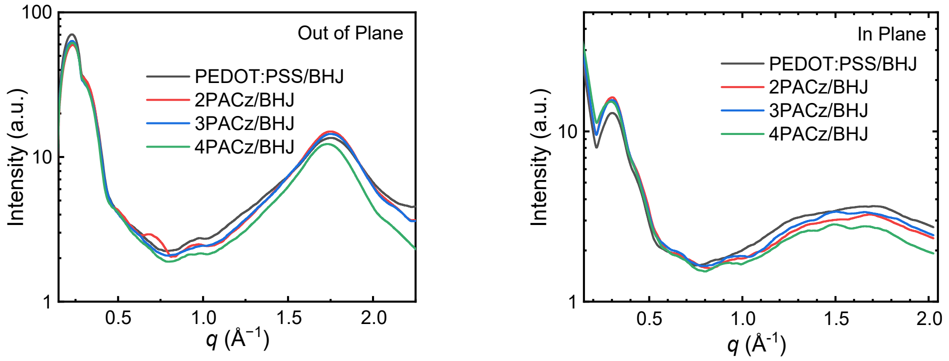

Figure S10. GIWAXS line cuts in the out of plane and in plane direction.

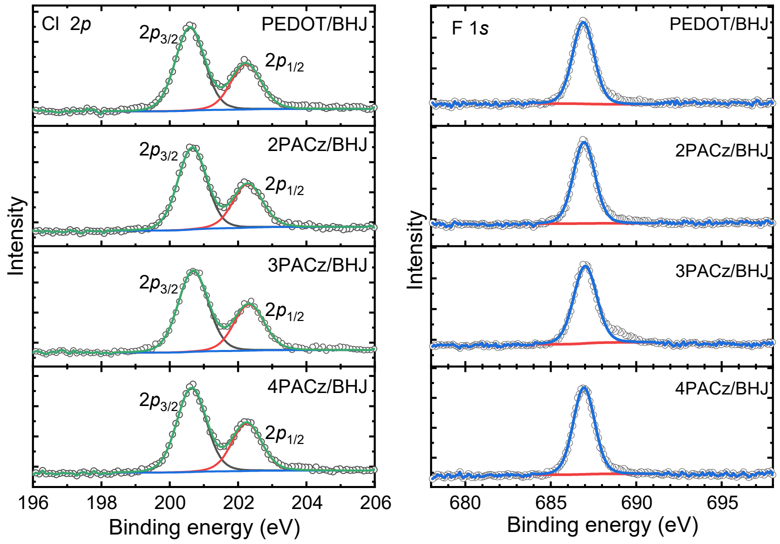

Figure S11. High-resolution XPS scans of the Cl 2*p* and F 1*s* atomic core levels for four active layers surface.

Table S6. Atomic concentration determined by XPS for in different BHJ layers.

Sample	C 1*s* (%At conc.)	Cl 2*p* (%At Conc.)	F 1*s* (%At Conc.)	N 1*s* (%At Conc.)	O 1*s* (%At Conc.)	S 2*p* (%At Conc.)
PEDOT/BHJ	80.38	1.13	1.86	2.57	6.45	7.61
2PACz/BHJ	80.73	1.04	1.85	2.50	6.26	7.62
3PACz/BHJ	80.36	1.13	1.93	2.55	6.76	7.52
4PACz/BHJ	80.84	1.08	1.72	2.10	6.51	7.50

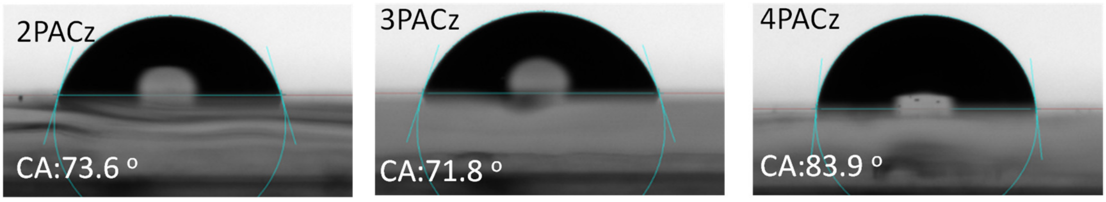

Figure S12. Water contact angles for 2PACz, 3PACz, and 4PACz.

Appendix ¹H NMR spectra and GC-MS/MALDI-TOF spectra of compounds

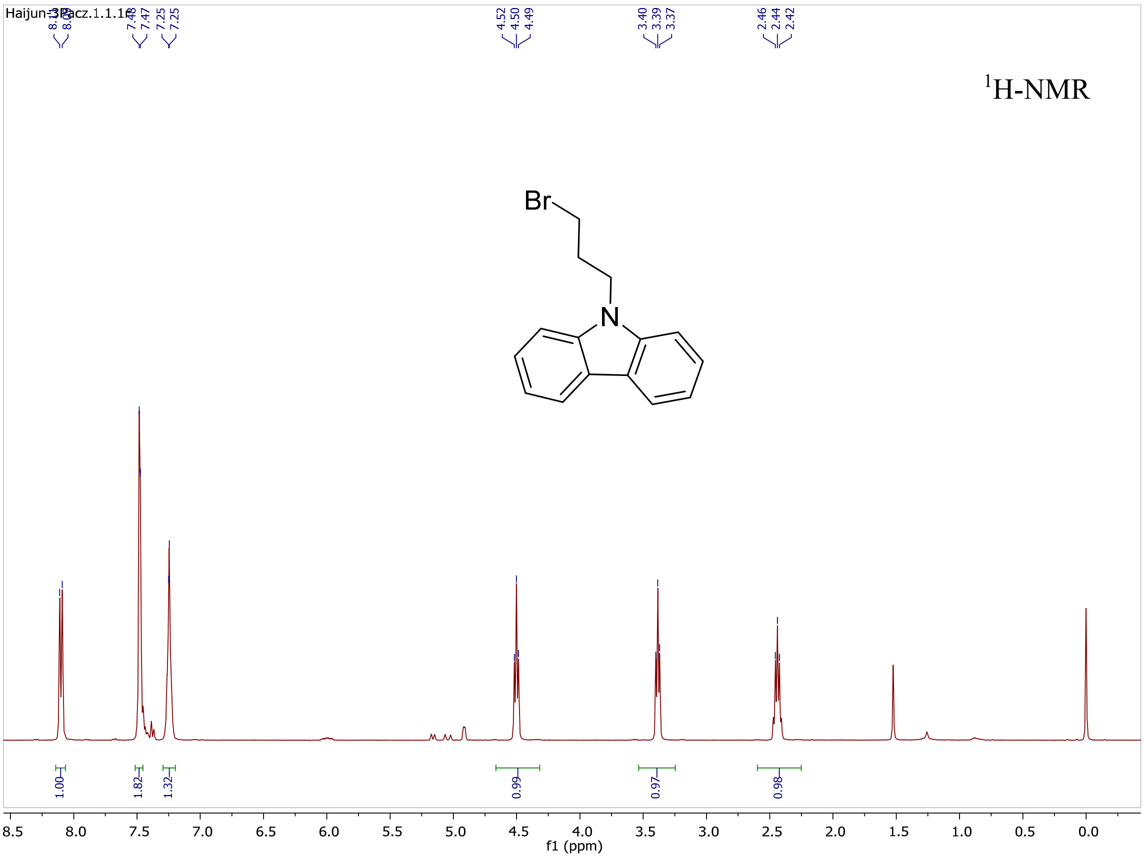

Spectrum S1. The ¹H NMR spectrum of compound **2b**.

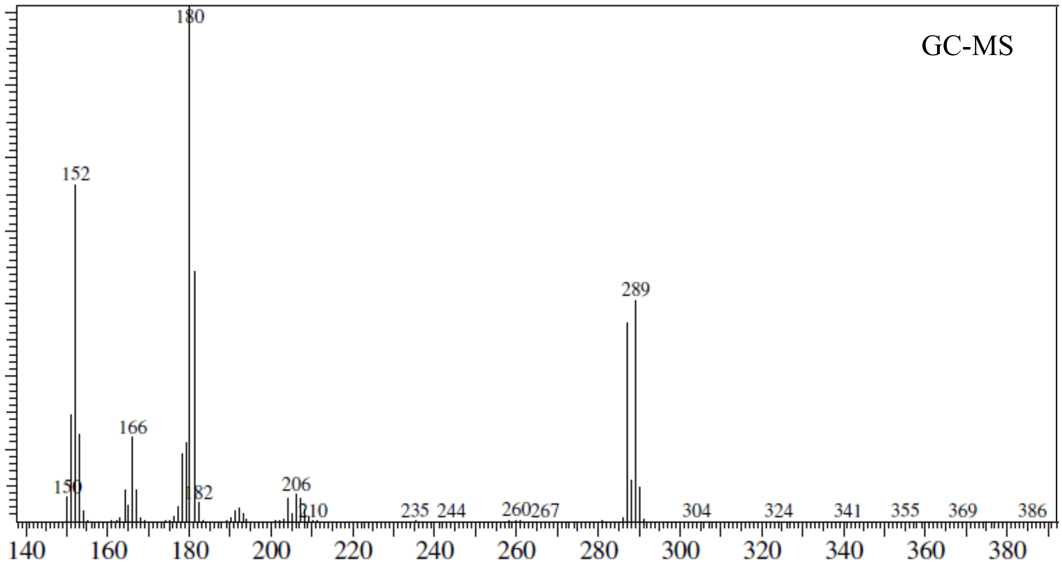

Spectrum S2. The GC-MS spectrum of compound **2b**.

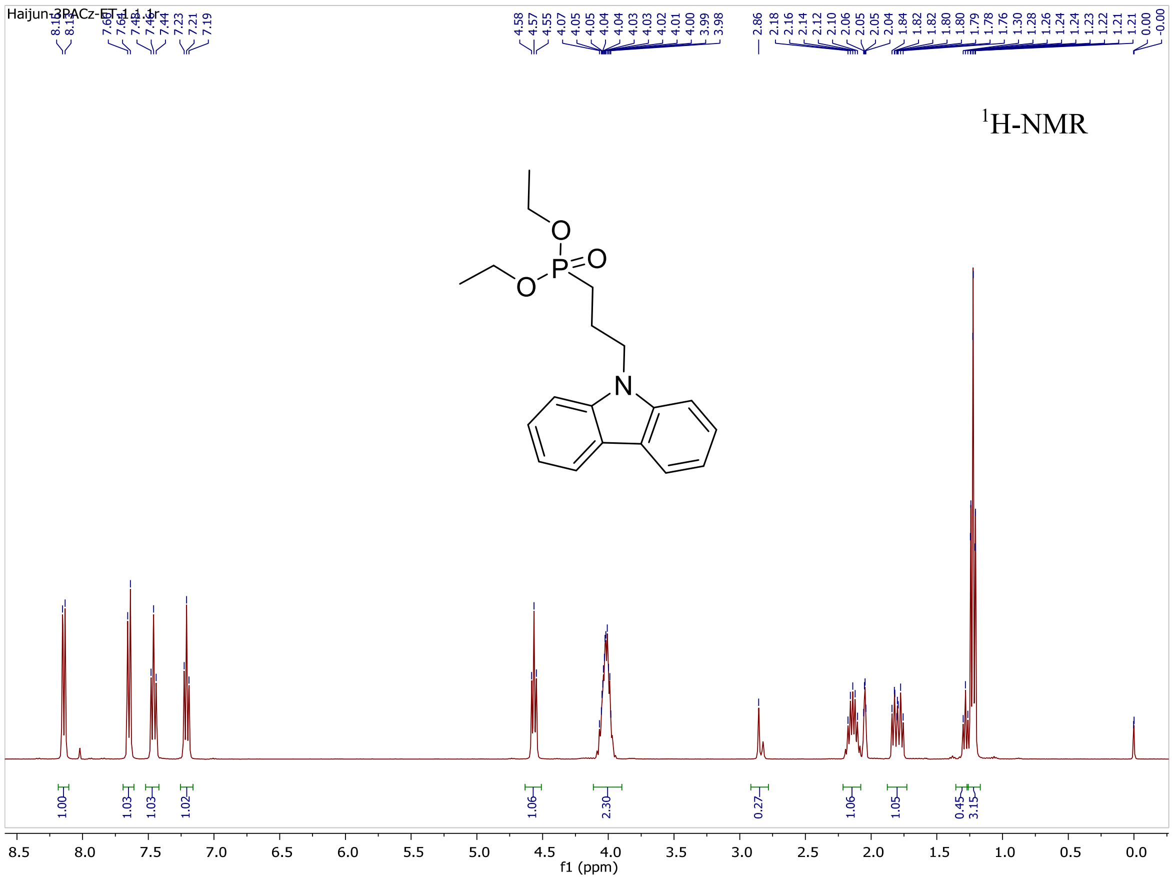

Spectrum S3. The ^{1}H NMR spectrum of compound **3b**.

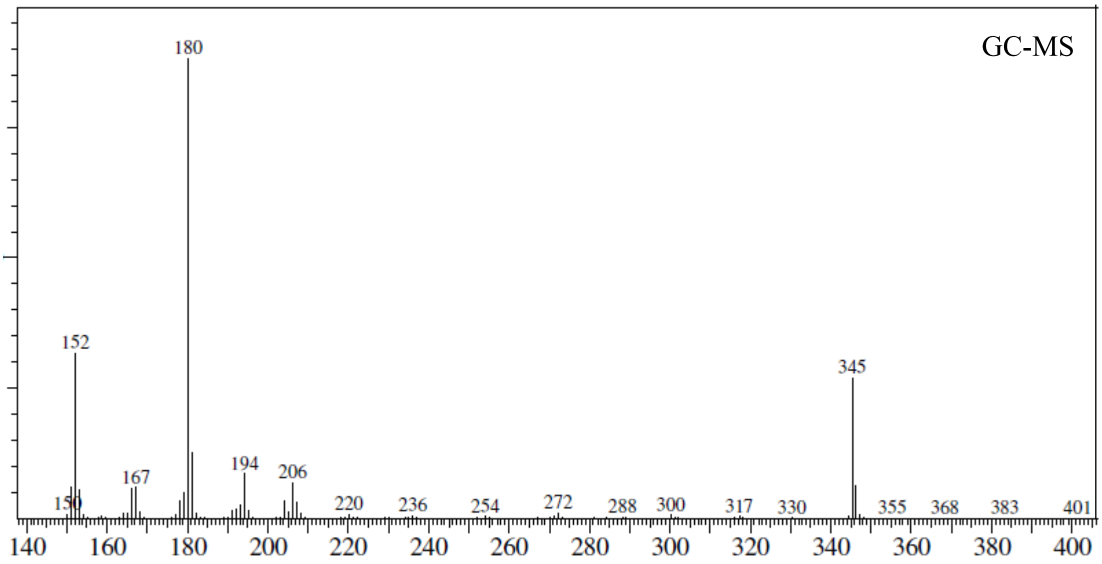

Spectrum S4. The GC-MS spectrum of compound **3b**.

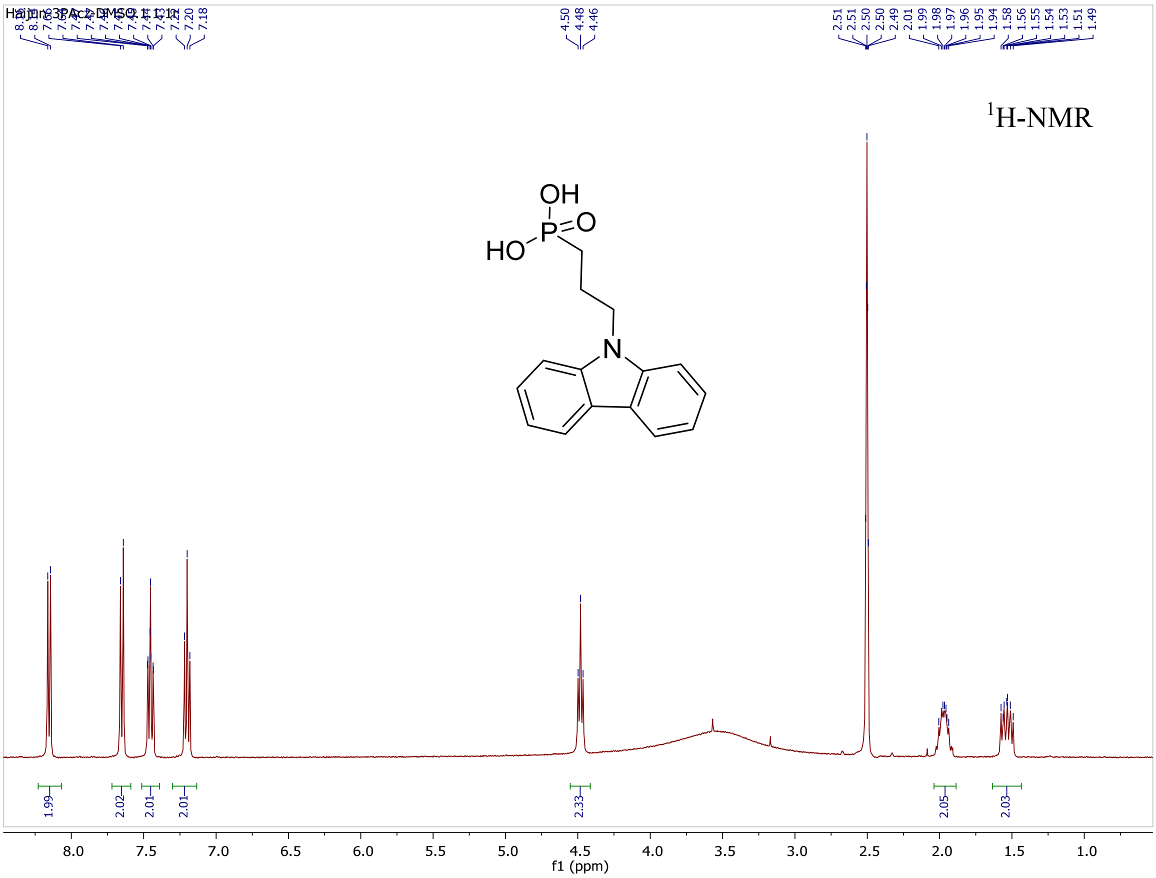

Spectrum S5. The ^{1}H NMR spectrum of 3PACz.

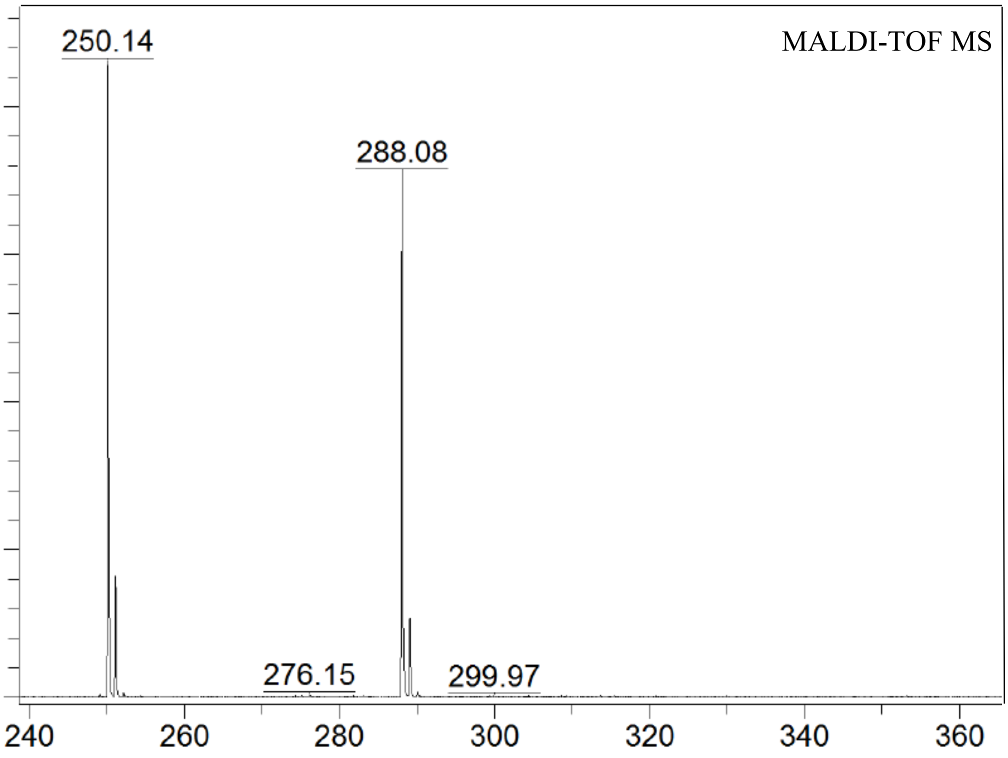

Spectrum S6. The MALDI-TOF MS spectrum of 3PACz.

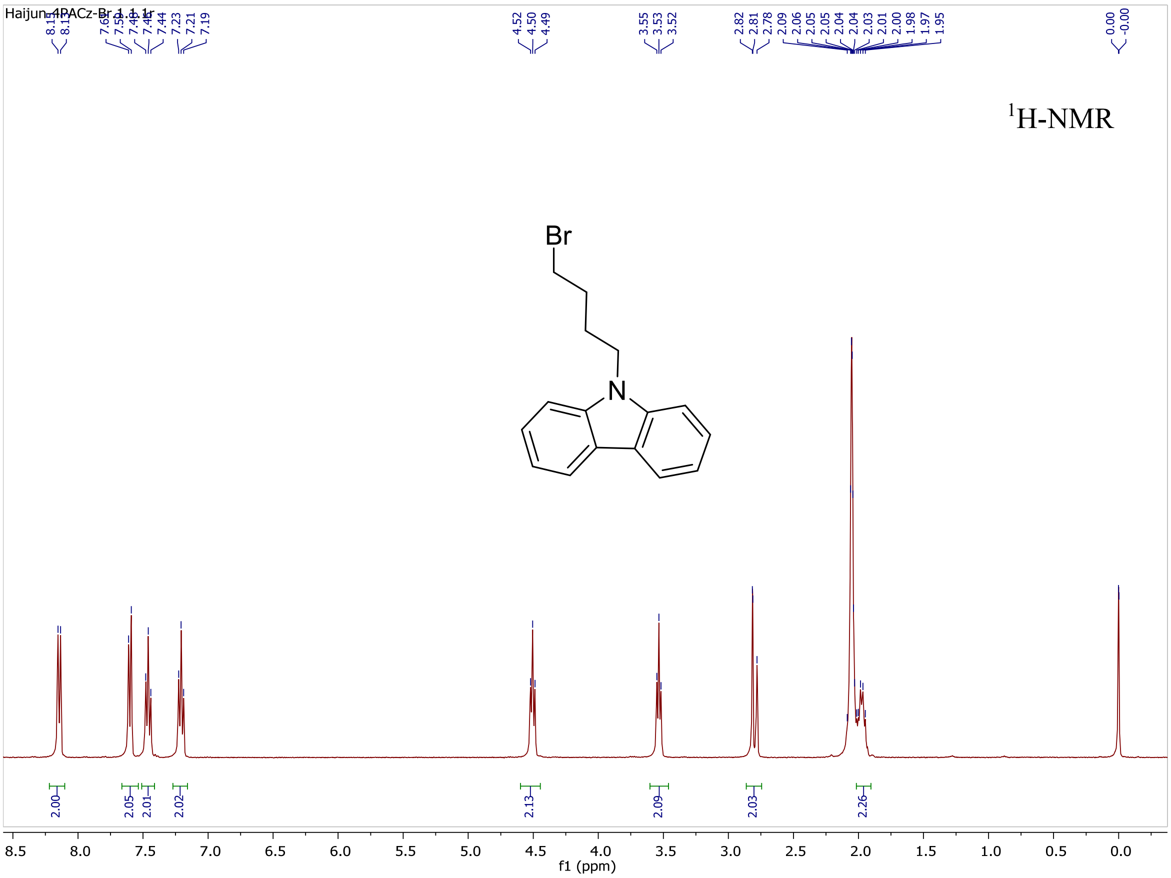

Spectrum S7. The ^{1}H NMR spectrum of compound **2c**.

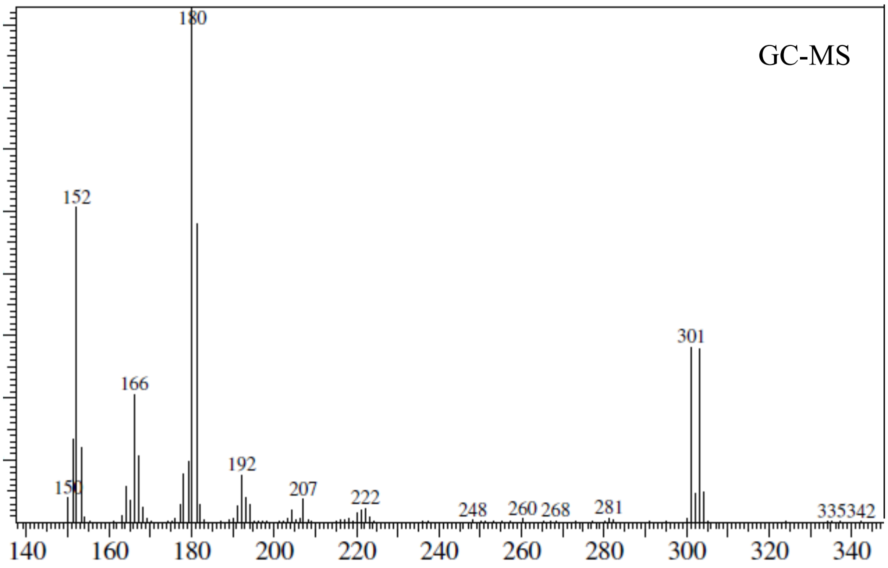

Spectrum S8. The GC-MS spectrum of compound **2c**.

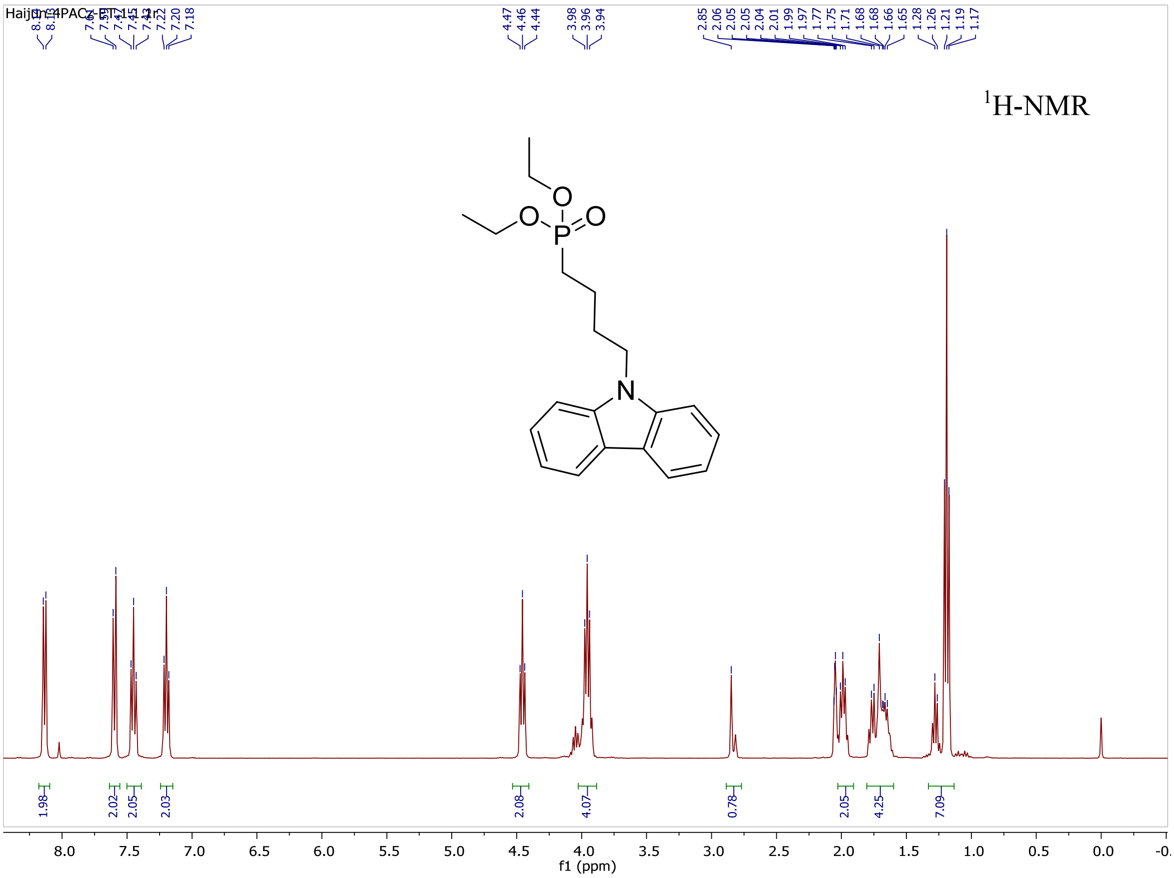

Spectrum S9. The ^{1}H NMR spectrum of compound **3c**.

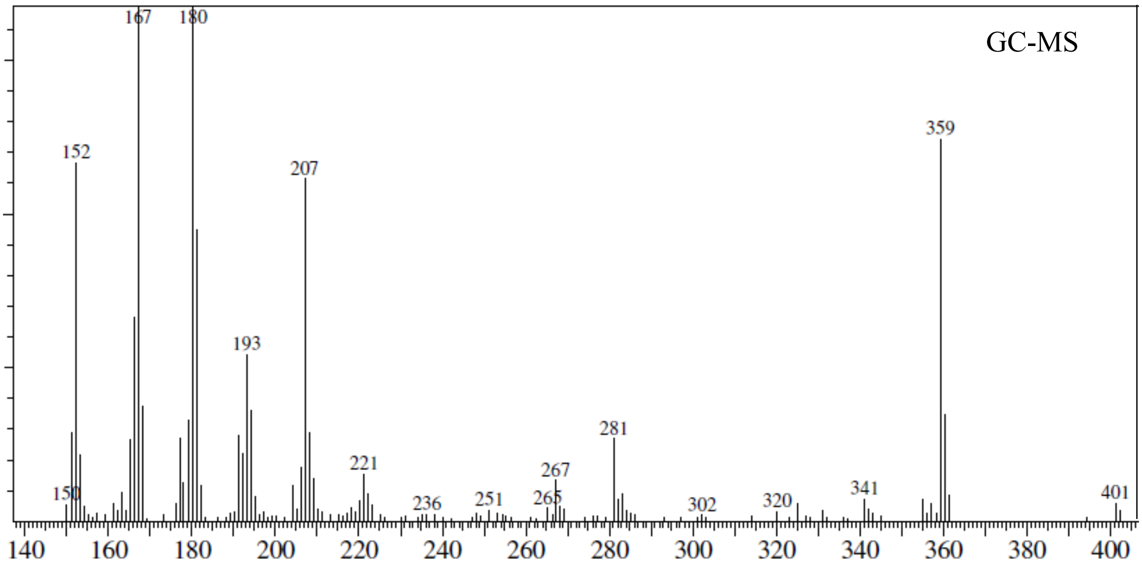

Spectrum S10. The GC-MS spectrum of compound **3c**.

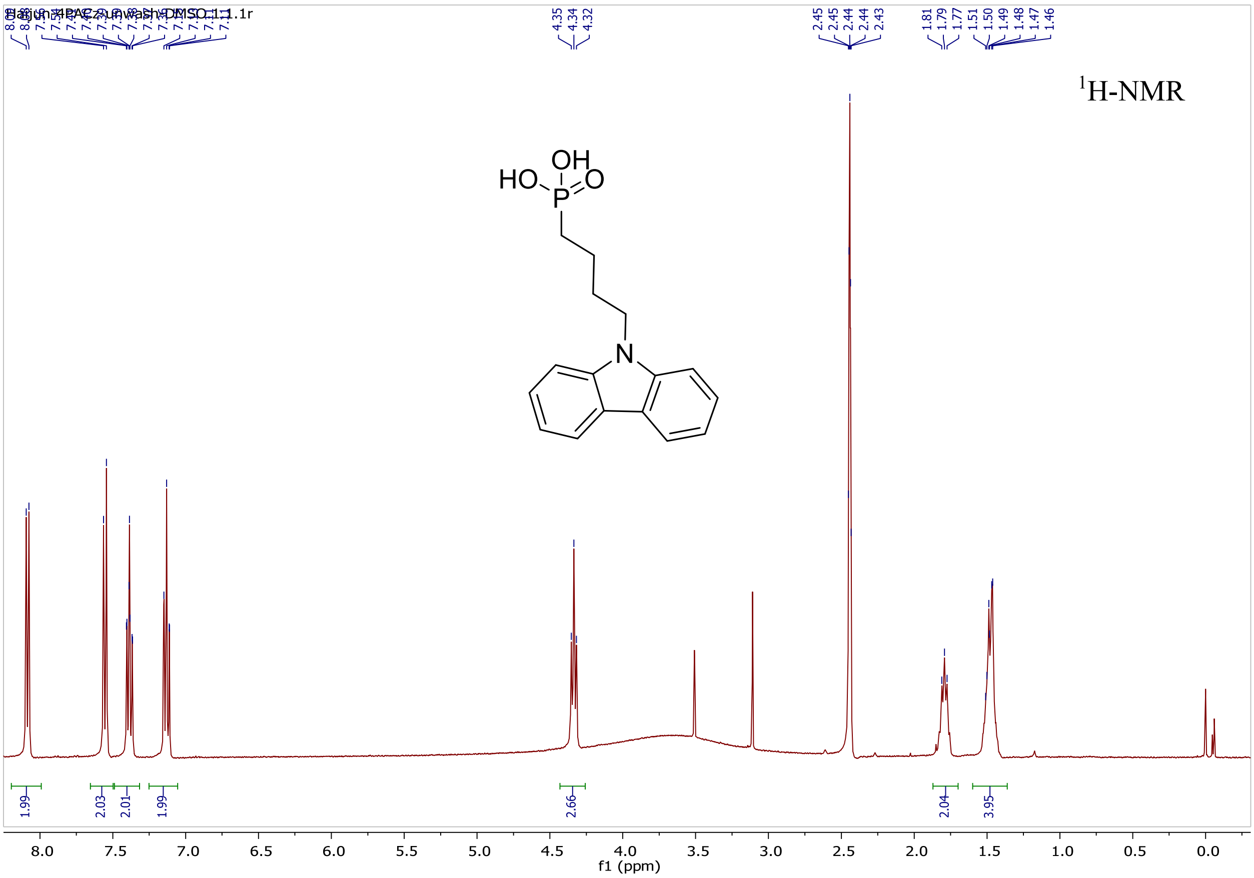

Spectrum S11. The ^{1}H NMR spectrum of 4PACz.

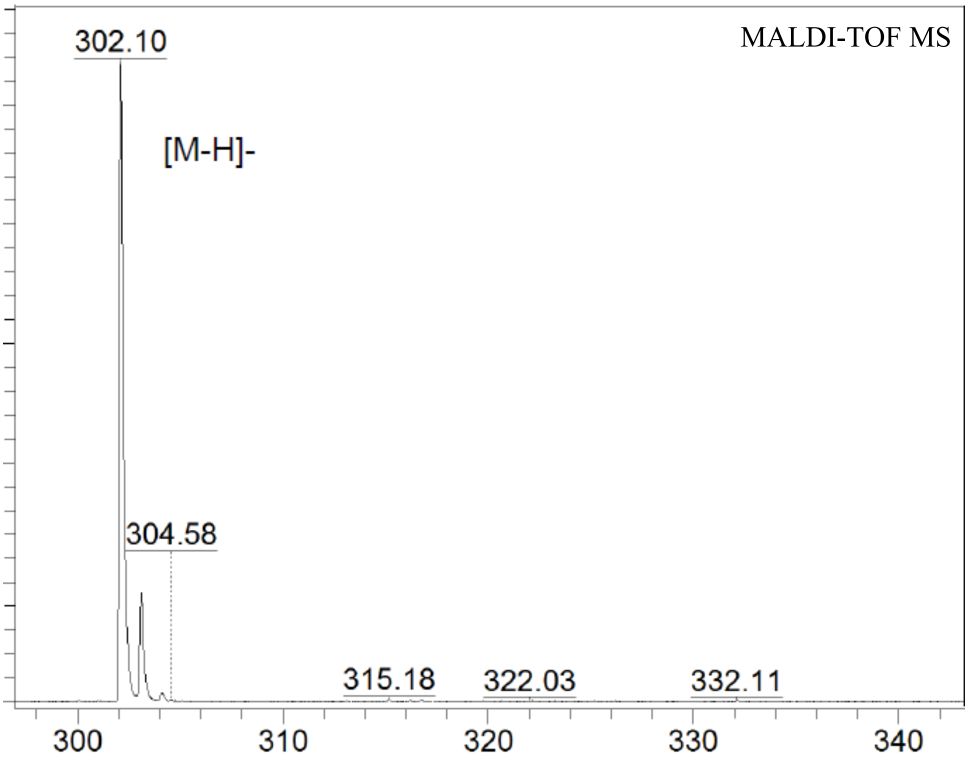

Spectrum S12. The MALDI-TOF MS spectrum of 4PACz.

Additional References

[1] Al-Ashouri, A.; Köhnen, E.; Li, B.; Magomedov, A.; Hempel, H.; Caprioglio, P.; Márquez, J. A.; Vilches, A. B. M.; Kasparavicius, E.; Smith, J. A.; Phung, N.; Menzel, D.; Grischek, M.; Kegelmann, L.; Skroblin, D.; Gollwitzer, C.; Malinauskas, T.; Jošt, M.; Matič, G.; Rech, B.; Schlatmann, R.; Topič, M.; Korte, L.; Abate, A.; Stannowski, B.; Neher, D.; Stolterfoht, M.; Unold, T.; Getautis, V.; Albrecht, S. Monolithic perovskite/silicon tandem solar cell with >29% efficiency by enhanced hole extraction. *Science* **2020**, 370, 1300−1309.

[2] Di Carlo Rasi, D.; Hendriks, K. H.; Wienk, M. M.; Janssen, R. A. J. Quadruple junction polymer solar cells with four complementary absorber layers. *Adv. Mater.* **2018**, *30*, 1803836.

[3] König, T. A. F.; Ledin, P. A.; Kerszulis, J.; Mahmoud, M. A.; El-Sayed, M. A.; Reynolds, J. R.; Tsukruk, V. V. Electrically Tunable Plasmonic Behavior of Nanocube–Polymer Nanomaterials Induced by a Redox-Active Electrochromic Polymer. *ACS Nano* **2014**, *8*, 6182−6192.

[4] Centurioni, E. Generalized matrix method for calculation of internal light energy flux in mixed coherent and incoherent multilayers. *Appl. Opt.* **2005**, *44*, 7532−7539.

[5] Palik, E. D. Handbook of optical constants of solids, Academic Press, **1991**.
